# Enhancing myocardial infarction detection with vectorcardiography: fusion-based comparative analysis of machine learning methods

**DOI:** 10.3389/fphys.2025.1683956

**Published:** 2026-01-05

**Authors:** Jaroslav Vondrak, Marek Penhaker

**Affiliations:** Faculty of Electrical Engineering and Computer Science, VSB-Technical University of Ostrava, Ostrava, Czechia

**Keywords:** myocardial infarction, vectorcardiography, electrocardiography, domain knowledge features dataset, ensemble learning, meta classifier

## Abstract

**Background:**

Early detection and diagnosis of myocardial infarction (MI) help physicians save lives through timely treatment. Vectorcardiography (VCG) is an alternative to the 12-lead electrocardiography, providing not only characteristic changes in cardiac electrical activity in MI patients but also unique spatial information often overlooked by traditional methods. Despite its potential, comprehensive comparative studies applying machine learning (ML) techniques specifically to VCG data remain limited.

**Methods:**

This study proposes a novel VCG processing methodology using a comparative analysis of machine learning-based algorithms for the automated detection of MI patients from VCG recordings, utilizing extracted domain knowledge VCG features that monitor morphological changes in cardiac activity. For this purpose, records from the PTB Diagnostic dataset were used. The extracted domain knowledge dataset of morphological features was then fed into a diverse set of 210 machine learning configurations, including K-nearest neighbor, Support Vector Machine, Discriminant Analysis, Artificial Neural Network, Decision Tree, Random Forest, Naive Bayes, Logistic Regression, and Ensemble Methods. To further improve classification performance, we combined analyzed high-performing models using a stacking ensemble strategy, which integrates multiple base classifiers into a meta-classifier.

**Results:**

The stacking-based decision-level fusion achieved high accuracy of 95.55%, sensitivity of 97.70%, specificity of 86.25%, positive predictive value of 96.86%, negative predictive value of 89.61% and f1-score of 97.27%.

**Conclusion:**

The results demonstrate that decision-level fusion via stacking improves classification performance and enhances the reliability of MI detection from VCG recordings, supporting cardiologists in decision-making.

## 1 Introduction

Myocardial infarction (MI) is a heart disease caused by a blockage of a coronary artery, causing insufficient flow of oxygenated blood to the heart muscle. A frequent clinical manifestation is long-lasting chest pain, shortness of breath or dizziness of varying intensity depending on where exactly in the coronary artery basin the blockage occurred (Thygesen et al., 2007; Zhao et al., 2021). There are, however, known cases where MI can occur in some patients as a clinically silent event without obvious symptoms. This fact can cause more serious problems for patients in the future. The American Health Association estimates that approximately 750,000 residents of the United States have a heart attack each year, of which 210,000 have a recurrent heart attack (Mozaffarian et al., 2016; Acharya et al., 2017). This means that there is a serious risk that patients can have a silent heart attack, which has damage to the myocardium without any clinical symptoms. The result of this fact is a very high risk of mortality. Mortality due to MI is increasing every year, while the age of onset of MI is decreasing (Acharya et al., 2017; Amini et al., 2021; He et al., 2022).

The result of MI is irreversible damage to the myocardium and early diagnosis is necessary. Among non-invasive diagnostic methods, the 12-lead ECG is the most commonly used in clinical practice for diagnosing MI and other heart diseases. It measures the electrical activity of the heart using electrodes placed on the patient’s body. The diagnosis is made based on changes in the shape of the curve, rhythm, and other characteristics that correspond to a specific pathology. As part of automated detection, the ECG is analyzed using Machine Learning (M-L) methods to support diagnostics. Various machine learning techniques are used Hammad et al. (2022), such as K-nearest neighbor (KNN) (Sharma and Sunkaria, 2018), Ensemble bagged trees (Fatimah et al., 2021), Support vector machine (SVM) (Sharma and Sunkaria, 2018; Dhawan et al., 2012; Dohare et al., 2018), a combination of Fourier decomposition method (FDM) and SVM Fatimah et al. (2021) or Convolutional neural networks (CNN) (Śmigiel et al., 2021). The use of 12-lead ECG for MI detection has been studied by several authors using different methods (Acharya et al., 2017; Zhang et al., 2021; Sharma et al., 2018; Zeng et al., 2020; Barmpoutis et al., 2019; Han and Shi, 2019; Tadesse et al., 2021). Murat et al. (2021) extracted ECG features from lead II, which were subsequently input to nine different classification methods for arrhythmia detection. Using ECG features, the random forest classifier achieved the highest accuracy of 90.30%. Zhang et al. (2021) achieved a detection accuracy of 99.40% using the Tree Bagger classification to locate 11 kinds of MI. Acharya et al. (2017) achieved a detection accuracy of 93.53% from unfiltered ECG recordings using CNN. In a previous study (Sharma et al., 2018), they also dealt with the KNN classifier for extracted features. Another use of SVM for MI classification was used by Dohare et al. (2018), where their proposed model does not include pre-processing of signals in the form of filtering. Other processing options of ECG were presented by Weng et al. (2014) in the form of a combination of Principal Component Analysis (PCA) and polynomial approximation in the feature extraction phase of MI classification in order to increase the accuracy of the SVM classifier where they achieved accuracy of 98.07%. Machine learning methods were also used for ECG classification by Zhou and Tan (2020), where they proposed a combination of CNN and extreme learning machine (ELM). While Ripoll et al. (2016) derived a new CNN-based screening method to assess whether a patient should be referred to a cardiology service from ambulatory care or the emergency department.

**FIGURE 1:**
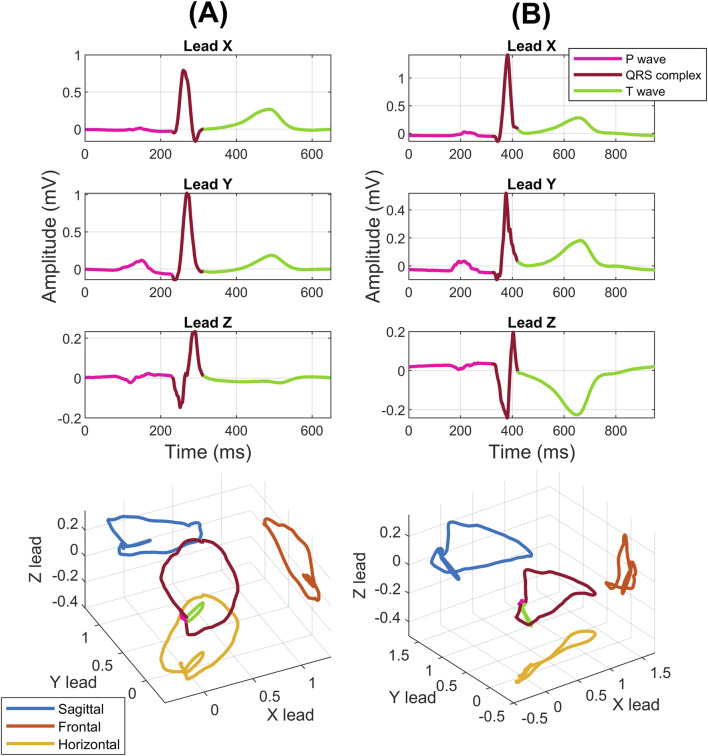
VCG recording for HC **(A)** and for MI **(B)** in individual leads X, Y, Z and three-dimensional recurrent loop.

In addition to the commonly used 12-lead ECG, there is also lesser-known method of 3-lead Vectorcardiography (VCG), which provides spatial information about the electrical activity of the heart. VCG measurement is performed in three mutually perpendicular anatomical planes: sagittal, horizontal and frontal. Figure 1 shows individual VCG leads X, Y, Z together with spatial visualization. Compared to a standard 12-lead ECG, VCG achieves higher sensitivity and is more suitable for automated processing to detect heart diseases due to the smaller number of leads, no redundant information and higher measurement accuracy with corrected orthogonal leads (Malmivuo and Plonsey, 1995; Kijonka et al., 2022). In clinical practice, vectorcardiography is most often measured using the Frank lead system (Frank, 1956). In addition to the Frank lead system, there are also other lead systems such as (McFee and Parungao, 1961), SVEC III (Schmitt and SIMONSON, 1955), and hybrid lead systems (Dellborg et al., 1995), which were only used in specialized workplaces.

Early detection of heart diseases is a frequently discussed topic these days (Hammad et al., 2018; Ketu and Mishra, 2022; Jambukia et al., 2015; Zhang X. et al., 2022, Zhang et al., 2020). There are known cases where automated detection from ECG recordings does not achieve sufficient accuracy (Filos et al., 2017). For example, studies dealing with the diagnosis of atrial enlargement (Bartall et al., 1978), right ventricular hypertrophy (Zhou et al., 2001; Cowdery et al., 1980) or even posterior MI (PMI) (Khan and Pachori., 2021a; Prabhakararao and Dandapat., 2020; Khan and Pachori., 2021b) found that VCG achieves a higher detection success compared to classical ECG. A reevaluation of the frequency of ECG use in favor of VCG has also been suggested (Van Bemmel et al., 1992; Khan and Pachori, 2021a). To detect MI from VCG recordings, the authors used similar principles of machine learning as in the case of ECG as mentioned above (Dehnavi et al., 2011; Dima et al., 2013; Panagiotou et al., 2013; Khan and Pachori, 2021a; Correa et al., 2016; Tripathy and Dandapat, 2017). For example, Dehnavi et al. (2011) extracted 22 VCG features, which were subsequently applied to a neural network (ANN). To detect myocardial scar as a consequence of MI, Dima et al. (2013) used ECG and VCG recordings from which they extracted features analyzing the pathophysiological consequences of a scarred myocardium causing electrical conduction failure. They used the SVM method for the extracted features. Another use of SVM was used by the authors in Panagiotou et al. (2013); Khan and Pachori (2021a) for the extracted features. The authors in Sharma and Sunkaria (2018) compared two classification methods for the detection of inferior MI, where they used the KNN and SVM methods and looked for which of these used methods achieves more accurate results. The information extracted from VCG led the authors in Chuang et al. (2023) to develop a patient-specific transformation for VCG synthesis. They used temporal convolutional networks in the variational mode decomposition domain to potentially differentiate MI patients and localize the infarct area. Perkins et al. (2024) used LASSO logistic regression to predict heart failure in patients after STEMI, using data from VCG and clinical information. Their model performed best at predicting heart failure within 90 days. They focused on important features that track changes in the heart’s shape, like the 3D sum or the shape of the heart in specific areas. In addition, the VCG recordings were also used in the analysis of the same MI patient before and during percutaneous transluminal coronary angiography (PTCA) (Hernandez et al., 2018). The results of this study indicate the potential for the diagnosis of acute myocardial infarction (AMI) using fewer leads.

### 1.1 Limitations of existing work

Machine learning methods have become an integral part of the automated processing of both ECG and VCG recordings for the early detection of various heart diseases. The limitation of the aforementioned publications is mainly the inconsistency of the analyzed machine learning methods for MI detection, where different authors achieve different detection accuracy results with different methods, even if many authors use similar features. Most authors do not dwell further on the choice or reason for using a given machine learning method. Furthermore, the spatial information of the VCG is not fully utilized in the processing of the measured signals. Another problem is the insufficient analysis of VCG features, especially within the framework of the analysis of the morphological properties of the given curve. A more detailed analysis of these features can contribute to a better understanding of the morphological changes that arise in individual patient records.

Comparison of different classification methods can increase the accuracy, reliability and efficiency of myocardial infarction detection and contribute to better care of patients suffering from this serious disease. The benefit of this study is the consolidation of knowledge in the processing of VCG records using machine learning methods for MI detection. These insights can further be beneficial in improving automated detection to support diagnostics. Furthermore, also for the subsequent selection of a suitable classifier when solving similar problems, which are often solved in the literature recently. The main novelty lies in the development of a unique methodology combining structured feature extraction, comparative analysis of multiple machine learning classifiers, and the final implementation of a stacking model that integrates their strengths. The key contributions of this work can be summarized as follows:

1. Extraction of domain-specific VCG features capturing morphological changes in VCG loops relevant to myocardial infarction.
2. Analysis of the contribution and relevance of VCG features using statistical and permutation importance of features analysis for MI detection.
3. Comparative analysis and tuning of various machine learning classifiers to assess their suitability for MI detection.
4. Enhancing MI detection performance through a novel methodology that integrates feature-based analysis and the fusion of machine learning models using a stacking approach to achieve higher accuracy in automated diagnosis.

### 1.2 Paper organization

The rest of the article is organized as follows: Section 2 describes the methods and data used with the analysis of extracted VCG features, Section 3 deals with the comparative analysis of individual machine learning methods within the MI detection accuracy, Section 4 focuses on the discussion of the achieved results and Section 5 concludes the paper.

## 2 Materials and methods

The main goal of this section is to design a methodology for improving the accuracy of MI detection from VCG recordings. The proposed methodology includes signal preprocessing, feature extraction for analyzing the morphological properties of VCG loops, feature relevance analysis for MI detection, and a comparative analysis of machine learning methods for automatic MI classification.

**FIGURE 2:**
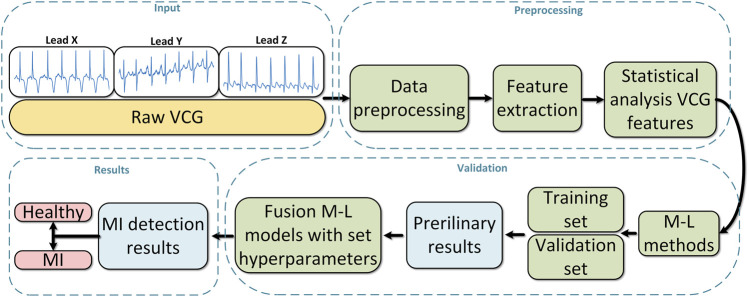
Block diagram of the sub-steps addressed in this work for the purpose of MI detection.

The individual sub-steps of processing VCG records for the purpose of MI detection are represented in Figure 2. Furthermore, each VCG record obtained from the Physikalisch Technische Bundesanstalt (PTB) database was filtered in order to remove interfering components that may affect the subsequent analysis. For this purpose, the procedures applied in previous work (Vondrak and Penhaker, 2022b) were used. After data preprocessing, VCG features were subsequently extracted, which analyze the morphological characteristics of cardiac activity. To verify the relevance of the extracted features, each feature was analyzed by the Mann-Whitney (M-W) statistical test to verify their predictive value. After statistical verification of significance, the features were fed into a total of 210 different types classification method configurations with variously set hyperparameters. The individual sub-steps mentioned above are discussed in more detail in the following subsections.

### 2.1 Study population

For the purposes of this study, a public PTB diagnostic database was used, which contains records from healthy patients and patients with various heart diseases (MI, cardiomyopathy, bundle branch block, dysrhythmia). Recordings were taken at the Department of Cardiology of University Clinic Benjamin Franklin in Berlin, Germany. Each recording is sampled at a frequency of 1 kHz and contains simultaneously measured 12-lead ECG and 3-lead VCG. Each patient record contain 15 simultaneously recorded signals: conventional 12-lead ECG and 3-lead Frank orthogonal VCG. The signals were acquired for 2 min with a 16-bit resolution in the range of ± 16.384 mV (Bousseljot et al., 1995; Goldberger et al., 2000). The following signal processing and applied machine learning methods for MI detection were analyzed in Matlab 2023a environment. For the purposes of this study, all 347 MI and 80 H C full-length recordings were used.

### 2.2 Data preprocessing

ECG and VCG recordings are often affected by disturbing components such as baseline wandering or high-frequency transients during measurement. This interference needs to be removed in order to obtain valid data from the desired biological signal. Since the data used mainly contain baseline wandering interference in all measured leads, a second-order Savitz-Golay (SG) filter with a window length of 1,201 was used. Unlike Butterworth or Chebyshev filters, the cut-off frequency of the SG filter is not strictly defined. Its value depends on the window length (window) and the filter order, which, according to Equation 1, is approximately 0.416 Hz.

$$f_{\mathrm{cut-off}}=\frac{1}{2\cdot window}\cdot f_{\mathrm{sampling}}$$ (1)

**FIGURE 3:**
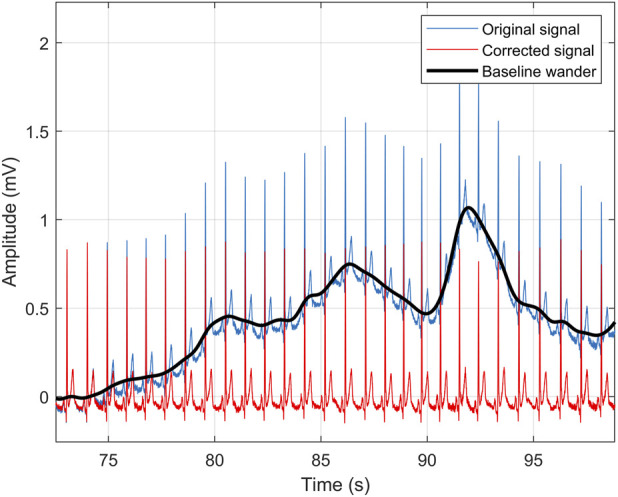
An illustrative example of signal filtering, where red curve represents the filtered signal, blue represents the original, and **black** represents the detected fluctuating component.

**FIGURE 4:**
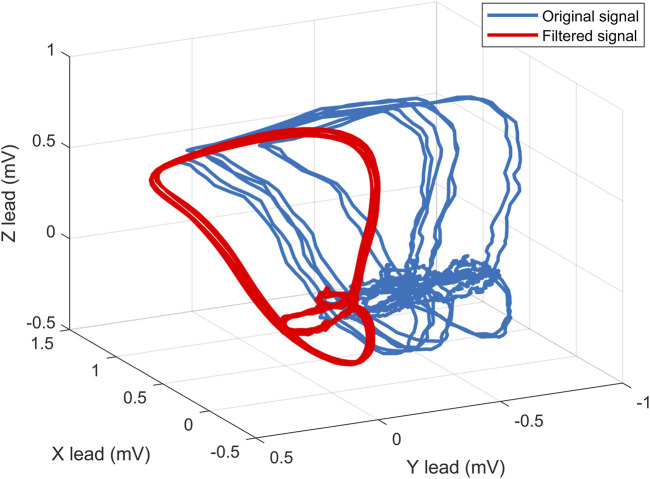
An example of spatial filtering of VCG loops, where the red curve is the filtered signal and the blue curve is the original signal.

This type of filtering is often used to remove the interfering component for subsequent analysis (Khan and Pachori, 2021b) and was also used in our previous works (Vondrak and Penhaker, 2022b). For illustrative purposes, Figure 3 shows the curve before filtering (blue), after filtering (red) and the fluctuating component (black). In addition, the effect of filtering on VCG spatial loops is shown in Figure 4. It can be noticed that this filter reliably removes interfering components in the signal that can affect the success of automatic detection.

### 2.3 Feature extraction

Based on our previous work, features extracted from VCG signals have proven effective for the detection of MI (Vondrak and Penhaker, 2022a; Vondrak et al., 2024). A total of 12 VCG features were used for the following analysis, analyzing the morphological properties of the QRS and T loop. These VCG features can be considered as mathematical operations applied to measured signals that do not require high computational requirements and can be applied to any recording. Furthermore, these features encapsulate domain knowledge, as they are derived from predefined characteristics based on specific insights into heart physiology (Rossello et al., 2013). Each of these extracted features was analyzed by a statistical analysis test using the Mann-Whitney test to verify their predictive value. The following features were extracted:

The length of the QRS curve (arcQRS): The length of the QRS loop was calculated from the projection to the optimal plane (OP) using the PCA method. This projection measures the total length of the loop with the ability to detect changes in the contour of the loop. The PCA method here represents the dimension reduction from a spatial loop to an optimal two-dimensional curve.

The principle of projection into the OP is based on the decomposition of the centered matrix $X_{c}$ , according to Equation 2, whose rows correspond to *N* samples of the QRS loop with zero mean. The columns of the centered matrix correspond to the three orthogonal leads.

$$X_{c}=U\cdot \Lambda \cdot W^{T}$$ (2)

where Λ is a diagonal N × 3 matrix of positive singular values, *U* is a real N × N matrix whose columns are orthogonal unit vectors of length *n*, referred to as the left singular vectors of the centered matrix *X*, and $W^{T}$ is a transposed 3×3 matrix whose columns are orthogonal unit vectors, referred to as the right singular vectors of the centered matrix *X*.

The columns of the matrix $W^{T}$ form a new orthogonal system into which the values of the matrix *X* are projected, known as the principal components, according to Equation 4. The first two vectors of the matrix *W*, together with the centroid *T* of the QRS loop, define the optimal plane ρ as given by Equation 3, which is specified parametrically.

ρ:A=T+t⋅w1+s⋅w2 (3)

where *A* is an arbitrary point of the optimal plane, *T* is a known point of the plane–the centroid of the QRS, *w1* and *w2* are the first two vectors of the matrix *W*, and *t* and *s* are the parameters of the optimal plane equation.

$$P=X_{c}\cdot W=U\cdot \Lambda$$ (4)

The first two principal components of the matrix *P* correspond to the projection of the loop points onto the optimal plane. The length of the curve can be expressed as shown in Equation 5.

$$arcQRS=\int \left(\sqrt{x_{\mathrm{PCA}}^{\prime }{\left(t\right)}^{2}+y_{\mathrm{PCA}}^{\prime }{\left(t\right)}^{2}dt}\right)$$ (5)

where $x_{\mathrm{PCA}}^{\prime }$ is the derivative of the first PCA component and $y_{\mathrm{PCA}}^{\prime }$ is the derivative of the second PCA component.

Maximum QRS and T loop vector (maxVecQRS, maxVecT): The maximum size of the vector in the QRS complex occurs during depolarization of the ventricles marked as the R wave. The QRS complex is followed by the T wave, which represents the repolarization of the ventricles. The maximum vectors for both loops were calculated from the maximum values in the given parts of the cardiac revolution (R peak, T peak) in individual leads X, Y, Z according to Equation 6.

$$maxVecQRS/T=\sqrt{X_{R/T}^{2}+Y_{R/T}^{2}+Z_{R/T}^{2}}$$ (6)

where $X_{R/T},Y_{R/T},Z_{R/T}$ represents maximum values in QRS or T loop in individual leads.

Maximum distance from the center of gravity (MaxGravQRS): The maximum distance from the centroid was calculated for individual points in the QRS loop according to Equation 7:

$$MaxGravQRS=max\left(\sqrt{{\left(X_{G}-X_{i}\right)}^{2}+{\left(Y_{G}-Y_{i}\right)}^{2}+{\left(Z_{G}-Z_{i}\right)}^{2}}\right)$$ (7)

where $X_{G}$ , $Y_{G}$ , $Z_{G}$ are coordinates of the center of gravity of the QRS loop. These values are obtained as average values in individual leads X, Y, Z and $X_{i}$ , ${\text{Y}}_{i}$ , ${\text{Z}}_{i}$ are individual points of the QRS loop.

Velocity of the QRS and T loop (VelQRS, VelT): The propagation velocity of the electrical vector was calculated for the QRS and T loop and can be obtained as the derivative of the curve *K* in time *t* according to the Equation 8.

$$VelQRS/T\left(t\right)=\frac{dK}{dt}$$ (8)

where *VelQRS/T(t)* is the velocity of the loop, *K* is the analyzed vectorcardiographic QRS or T loop and *t* is time. Additional features were then calculated from the velocity obtained this way, namely the maximum speed (maxVelQRS/T), mean velocity (meanVelQRS/T) and standard deviation of the velocity (stdVelQRS/T). Similarly, all of these features were also obtained for the T loop.

The area under QRS and T loop (areaQRS/T): The area under the QRS and T loops was calculated from the projection in the OP according to Equation 9. Area under the curve was calculated for both QRS and T loop.

$$areaQRS/T=\frac{1}{2}|\overset{n}{\underset{i=1}{\sum }}\left(x_{PCA1_{i}}+x_{PCA2_{i}}\right)\left(y_{PCA1_{i}}-y_{PCA2_{i}}\right)$$ (9)

where $x_{\mathrm{PCA}}$ is the first PCA component, $y_{\mathrm{PCA}}$ is the second PCA component and *i* is the number of samples of the first and second PCA.

### 2.4 Classifications

The classification methods most commonly used in the literature for distinguishing between MI patients and healthy individuals were selected. These include K-nearest neighbor (KNN) (Mucherino et al., 2009), Support Vector Machine (SVM) (Hearst et al., 1998), Discriminant Analysis (DA) (Balakrishnama and Ganapathiraju, 1998), Artificial Neural Network (ANN) (Dreiseitl and Ohno-Machado, 2002), Decision Tree (DT) (Mantas and Abellan, 2014), Random Forest (RF) (Belgiu and Dr Drăfuţ, 2016), Naive Bayes (NB) (Murphy, 2006), Logistic Regression (LR) (Ng and Jordan, 2001) or Ensemble Methods (EM) (Dietterich, 2000). However, several factors must be taken into account when analyzing individual classifiers. These factors include the nature of the data distribution, the variability of the data, and the diversity or existence of noise. It is also necessary to keep in mind the advantages and possible limitations of individual classifiers. For example, KNN must have training and test data available at all times, so the classifier relies on memory-based learning. For noisy data, the choice using DT is more preferred (Mantas and Abellán, 2014). Or for linear and non-linear features, SVM classifiers are effective (Noble, 2006).

In this study, we experimented with the above-mentioned classifiers using 10-fold cross-validation, while each of the used classifiers was tuned with respect to the relevant parameters. The DT has been tuned with respect to the split criterion (Cross entropy and Gini diversity index (GDI)), minimum number of leaves as $2^{n}$ (2, 4, 8, 16), and maximum number of decision splits (5, 10, 20, 50).

In the case of SVM, the classifier has been tuned with regard to kernels, namely Linear kernel, Polynomial kernel [with degree (2, 3, 4)], Radial Basis Function (RBF) kernel, and Gaussian kernel. A kernel scale from 1 to 5 was used for each of the kernels.

The KNN was tuned with respect to the size of K, which was chosen between 5 and 20 with a step of 5. The distance type was chosen as Euclidian, City block, and Chebyshev. A distance parameter was chosen Equal, Inverse, and Squared inverse.

DA has been tuned with respect to the type of discriminant analysis, namely linear, diagonal linear, pseudo-linear, quadratic, diagonal quadratic and pseudo-quadratic.

The ANN has been tuned by adjusting both the number of hidden layers (ranging from 1 to 2 layers) and the size of each hidden layer (with units set at 5, 10, 20, and 50 neurons). This tuning is focused on finding the right balance between model complexity and generalization, helping the network better capture complex patterns in the data.

The NB has been tuned with respect to the Prior (uniform, empirical), distribution (normal, kernel) and in the case of distribution kernel the kernel type as normal, box, epanechnikov and triangle.

The Random forests are based on a similar principle as Decision Trees. For this method, the parameters that were tuned included the Number of Trees, ranging from 100 to 500 in steps of 100, and the Minimum Number of Leaves, ranging from 1 to 5 in steps of 1. Higher values were not considered due to the risk of potential overfitting.

The Logistic Regression was tuned with the Prior parameter set to uniform and empirical, which influences how class weights are assigned in the training data. For Regularization, Least Absolute Shrinkage and Selection Operator (lasso) and ridge methods were selected to prevent overfitting by penalizing overly complex models. Additionally, various optimization algorithms (Solver) such as Stochastic gradient descent (sgd) (Shalev-Shwartz et al., 2007), Average stochastic gradient descent (asgd) (Xu, 2011), Dual sgd for SVM (dual) (Hsieh et al., 2008), Broyden-Fletcher-Goldfarb-Shanno quasi-Newton algorithm (bfgs) (Nocedal and Wright, 1999), Limited-memory bfgs (lbfgs) (Nocedal and Wright, 1999), and Sparse Reconstruction by Separable Approximation (sparsa) (Wright et al., 2009) were tested to find the optimal model parameters.

Next, the Ensemble Methods were tuned with regard to different techniques: Adaptive Boosting, which enhances weak learners by focusing on misclassified examples; Robust Boosting, designed to handle noisy data by adjusting the impact of outliers; and Random Undersampling Boosting, which balances class distribution by reducing the size of the majority class. The Learner type was set to either Tree or Discriminant, depending on the model’s requirements. Learning cycles were tested with values of 50, 100, 150, and 200 to optimize the model’s performance.

According to the aforementioned hyperparameter settings, a total of 210 different configurations of machine learning methods are analyzed. These hyperparameters were chosen with consideration for preventing overfitting of individual classifiers, computational complexity, and appropriate optimizations for each model.

Finally, the Stacking ensemble learning method was used for classification, combining predictions from multiple base models to enhance classification performance. The input for logistic regression as a meta-classifier will consist of the best-performing trained models. Stacking ensemble learning is a machine learning method that combines the outputs of multiple base models and utilizes a meta-classifier to achieve better performance (Alexandropoulos et al., 2019; Yoon and Kang, 2023; Zhou, 2025). Each base model learns from the data from different perspectives, reducing the risk of overfitting and improving generalization. The meta-classifier, often logistic regression, is trained on the outputs of these models and learns to weigh their predictions based on their accuracy. This approach represents a form of decision-level fusion, as it integrates multiple model predictions to leverage the strengths of different algorithms and increase the overall robustness of the classification. The result is a model with higher accuracy and reliability compared to individual classifiers. To prevent data leakage and ensure that the meta-classifier learned only from unseen samples, the stacking layer was trained using out-of-fold (OOF) predictions obtained during cross-validation. Each base model produced predictions exclusively for data that were not part of its training fold. These predictions were subsequently used as input features for training the meta-classifier.

### 2.5 Performance parameters

In this study, the performance of the classifiers was evaluated by calculating accuracy (Acc) (Equation 10), sensitivity (Sens) (Equation 11), specificity (Spec) (Equation 12), positive predictive value (PPV) (Equation 13), negative predictive value (NPV) (Equation 14), and f1-score (f1) (Equation 15). Whereby TN (True Negative) represents the number of correctly detected healthy records. TP (True Positive) represents the number of correctly detected MI records. FN (False Negative) represents the number of false-negative samples, i.e. the number of MI records that were not correctly detected. FP (False Positive) represents the number of false-positive samples, i.e. the number of healthy records that were incorrectly detected as MI.

$$Sens=\frac{TP}{TP+FN}$$ (10)

$$Spec=\frac{TN}{TN+FP}$$ (11)

$$PPV=\frac{TP}{TP+FP}$$ (12)

$$NPV=\frac{TN}{TN+FN}$$ (13)

$$Acc=\frac{TN+TP}{TN+TP+FN+FP}$$ (14)

$$f1=\frac{2\cdot PPV\cdot Sens}{PPV+Sens}$$ (15)

In addition to the above parameters a confusion matrix is also included, which gives a count of how well the classification model works by showing the number of correct and incorrect predictions for each class. It allows distinguishing between true positive, false positive, true negative and false negative predictions, which is key to evaluating model performance.

## 3 Results

The VCG signals in individual leads and the three-dimensional loop corresponding to HC and MI can be seen in Figure 1. From Figure 1, morphological changes are evident both in individual leads and in the spatial display. These morphological changes indicate the non-stationarity of the VCG signal. In this study, we used VCG features to analyze these morphological changes. We extracted a total of 12 VCG features that focus on morphological changes in both the QRS and the T segment.

### 3.1 Relevance of VCG features

To verify the informative value of individual features, each feature was tested with the M-W statistical test, due to the fact that for each feature we are dealing with two-sample data (MI and HC), where a statistically significant difference is sought. The use of M-W statistical test was expedient, because according to the Shapiro-Wilk (S-W) test, the data do not come from a normal distribution, the p-values of the S-W test for all VCG features are ≪ 0.05. The following hypotheses were analyzed for the S-W test:

$H_{0}$ : The data are samples from a normal distribution.

$H_{A}$ : $\neg H_{0}$ (negation of the null hypothesis)

The results of the M-W test confirmed the significance (p-value ≪ 0.05) of individual features for distinguishing the analyzed MI and HC groups. The results of the S-W and M-W test with p-values for individual features can be seen in Table 1. The following hypotheses were analyzed for the M-W test:

$H_{0}$ : $MI_{0,5}$ = $HC_{0,5}$ (There are no statistically significant differences between the median MSE of MI and HC.)

$H_{A}$ : $\neg H_{0}$ (negation of the null hypothesis)

**TABLE 1:** The p-values for the 12 extracted features dividing records into MI and HC groups.

No.	VCG feature	S-W p-value	M-W p-value
1	Std velocity T	$6.8\cdot 10^{-10}$	$2.4\cdot 10^{-17}$
2	Area QRS	$2.6\cdot 10^{-13}$	$1.7\cdot 10^{-15}$
3	Std velocity QRS	$6.1\cdot 10^{-15}$	$7.4\cdot 10^{-14}$
4	Max vector T	$3.3\cdot 10^{-16}$	$1.3\cdot 10^{-13}$
5	Length of QRS	$4.9\cdot 10^{-17}$	$8.2\cdot 10^{-13}$
6	Max velocity QRS	$4.8\cdot 10^{-18}$	$1.3\cdot 10^{-10}$
7	Mean velocity T	$2.4\cdot 10^{-11}$	$6.2\cdot 10^{-10}$
8	Mean velocity QRS	$3.7\cdot 10^{-14}$	$4.8\cdot 10^{-9}$
9	Max velocity T	$1.9\cdot 10^{-13}$	$7.9\cdot 10^{-9}$
10	Max grav distance QRS	$5.6\cdot 10^{-12}$	$1.7\cdot 10^{-8}$
11	Max vector QRS	$4.3\cdot 10^{-9}$	$2.3\cdot 10^{-8}$
12	Area T	$6.9\cdot 10^{-11}$	$1.7\cdot 10^{-6}$

Clinically, the observed differences in VCG features reflect alterations in the processes of ventricular depolarization and repolarization that occur as a result of ischemic injury. The lower T-loop velocity and smaller QRS loop area found in MI patients indicate delayed or weakened propagation of electrical activity within the infarcted myocardium. These findings suggest that the extracted VCG features not only provide statistically significant discrimination between HC and MI recordings but also have clear physiological relevance, supporting their use in automated MI detection.

**FIGURE 5:**
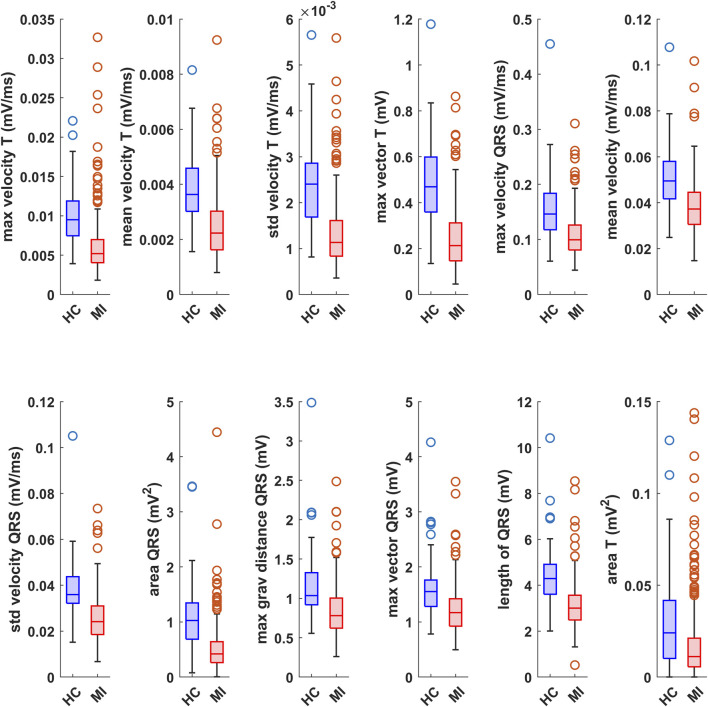
Boxplots of the values of individual VCG features for the analyzed MI and HC records, where the red boxes belong to MI and the blue boxes belong to HC.

A similar comparison is shown in Figure 5, where boxplots of individual features for the MI and HC groups are shown. We prioritized features for which the distributions of the MI and HC groups showed minimal overlap. For each group, there are also outlier observations that are usually removed from the observed dataset in common statistical tests. However, these values were not removed because they are real data that can occur in clinical practice. Since all the extracted VCG features show a significant ability to distinguish the analyzed groups, we came to the conclusion that for further processing all 12 features will be fed into the classification phase. The relevance of individual features can be seen in Table 1, sorted according to the p-value of the M-W test, while the most relevant feature is the one with the lowest p-value. Since all features show a result of statistical tests lower than the level of significance α , all VCG features are used for subsequent analyses.

### 3.2 Analysis of classification methods

The results of individual classification methods are shown in Tables 3–10. However, it is important to note that we used a 10-fold cross-validation analysis. This approach helps to avoid overfitting due to data imbalance and the inclusion of multiple segments from the same patient, which can bias the results. To determine the best results, accuracy is used as the primary performance metric for each classification method. The setting that achieves the highest accuracy is highlighted in the corresponding tables.

In the literature, the Support Vector Machine method is frequently used for classification problems due to its ability to work with various kernel types and effectively separate nonlinearly separable data classes. In this analysis, different SVM classifier configurations with various hyperparameters were tested, and their results are summarized in Table 2. The best performance was achieved with a polynomial kernel of order 3 and a scale parameter set to 1, reaching the highest accuracy of 91.10%.

**TABLE 2:** Performance of Support Vector Machine classifier with different set hyperparameters using 10-fold cross-validation.

Kernel	Scale	Sens	Spec	Acc	PPV	NPV	f1
Polynomial order 2	1	0.9568	0.6125	0.8923	0.9146	0.7656	0.9353
	2	0.9683	0.4625	0.8735	0.8865	0.7708	0.9255
	3	0.9712	0.4250	0.8689	0.8799	0.7727	0.9235
	4	0.9769	0.3875	0.8665	0.8737	0.7949	0.9232
	5	0.9798	0.2125	0.8361	0.8437	0.7083	0.9068
Polynomial order 3	**1**	**0.9510**	**0.7375**	**0.9110**	**0.9402**	**0.7763**	**0.9456**
	2	0.9683	0.5125	0.8829	0.8960	0.7885	0.9305
	3	0.9712	0.4500	0.8735	0.8845	0.7826	0.9258
	4	0.9683	0.4375	0.8689	0.8819	0.7609	0.9238
	5	0.9769	0.4250	0.8735	0.8805	0.8095	0.9259
Polynomial order 4	1	0.9424	0.7125	0.8993	0.9343	0.7403	0.9383
	2	0.9568	0.6750	0.9040	0.9274	0.7826	0.9419
	3	0.9712	0.4750	0.8782	0.8892	0.7917	0.9285
	4	0.9712	0.4250	0.8689	0.8799	0.7727	0.9244
	5	0.9741	0.4250	0.8712	0.8802	0.7907	0.9258
Linear	1	0.9683	0.4625	0.8735	0.8865	0.7708	0.9255
	2	0.9654	0.3625	0.8525	0.8679	0.7073	0.9134
	3	0.9769	0.1250	0.8173	0.8289	0.5556	0.8974
	4	0.9914	0.0500	0.8150	0.8190	0.5714	0.8989
	5	0.9942	0.0375	0.8150	0.8175	0.6000	0.8987
RBF	1	0.9597	0.5250	0.8782	0.8976	0.7500	0.9277
	2	0.9568	0.4250	0.8571	0.8783	0.6939	0.9160
	3	0.9625	0.4125	0.8595	0.8766	0.7174	0.9186
	4	0.9625	0.3625	0.8501	0.8675	0.6905	0.9141
	5	0.9741	0.2250	0.8337	0.8450	0.6667	0.9077

Performance of Support Vector Machine classifier with experimentally adjusted hyperparameters with the best result marked in bold.

The results for individual types of discriminant analysis are shown in Table 3, where linear discriminant analysis (LDA) achieves the most accurate results with accuracy of 90.40%. The accuracy value is similar to the DT classifier, however, with different sensitivity and specificity values.

**TABLE 3:** Performance of Discriminant Analysis classifier with different set hyperparameters using 10-fold cross-validation.

Discriminant type	Sens	Spec	Acc	PPV	NPV	f1
**Linear**	**0.9510**	**0.7000**	**0.9040**	**0.9322**	**0.7671**	**0.9415**
Diag. Linear	0.8905	0.6750	0.8501	0.9224	0.5870	0.9062
Pseudolinear	0.9481	0.7000	0.9016	0.9320	0.7568	0.9399
Quadratic	0.9308	0.7250	0.8923	0.9362	0.7073	0.9335
Diag. Quadratic	0.8934	0.6250	0.8431	0.9118	0.5747	0.9025
Pseudoquadratic	0.9308	0.7500	0.8970	0.9417	0.7143	0.9361

Performance of Discriminant Analysis classifier with experimentally adjusted hyperparameters with the best result marked in bold.

Table 4 shows the results of the KNN classification method for experimentally set hyperparameters. KNN setting K = 10, distance type City block and distance parameter Squared inverse achieved the most accurate results, where it achieved a detection accuracy of 89.93%.

**TABLE 4:** Performance of KNN classifier with different set hyperparameters using 10-fold cross-validation.

Number of neighbors	Type of distance	Distance parameter	Sens	Spec	Acc	PPV	NPV	f1
K = 5	Euclidian	Equal	0.9308	0.6750	0.8829	0.9255	0.6923	0.9281
		Inverse	0.9251	0.7125	0.8852	0.9331	0.6867	0.9291
		Squared inverse	0.9222	0.7125	0.8829	0.9329	0.6786	0.9274
	City block	Equal	0.9251	0.7250	0.8876	0.9359	0.6905	0.9304
		Inverse	0.9251	0.7500	0.8923	0.9413	0.6977	0.9328
		Squared inverse	0.9280	0.7375	0.8923	0.9388	0.7024	0.9333
	Chebyshev	Equal	0.9280	0.5625	0.8595	0.9020	0.6429	0.9151
		Inverse	0.9280	0.6375	0.8735	0.9174	0.6711	0.9226
		Squared inverse	0.9280	0.6750	0.8806	0.9253	0.6835	0.9266
**K = 10**	Euclidian	Equal	0.9481	0.5875	0.8806	0.9088	0.7231	0.9280
		Inverse	0.9337	0.7000	0.8899	0.9310	0.7089	0.9323
		Squared inverse	0.9222	0.6500	0.8712	0.9195	0.6582	0.9208
	**City block**	Equal	0.9366	0.5625	0.8665	0.9028	0.6716	0.9194
		Inverse	0.9366	0.7000	0.8923	0.9312	0.7179	0.9337
		**Squared inverse**	**0.9366**	**0.7375**	**0.8993**	**0.9393**	**0.7284**	**0.9379**
	Chebyshev	Equal	0.9337	0.5375	0.8595	0.8975	0.6515	0.9152
		Inverse	0.9193	0.6750	0.8735	0.9246	0.6585	0.9219
		Squared inverse	0.9222	0.6500	0.8712	0.9195	0.6582	0.9208
K = 15	Euclidian	Equal	0.9308	0.6000	0.8689	0.9099	0.6667	0.9202
		Inverse	0.9366	0.6625	0.8852	0.9233	0.7067	0.9295
		Squared inverse	0.9366	0.7000	0.8923	0.9312	0.7179	0.9337
	City block	Equal	0.9308	0.5875	0.8665	0.9073	0.6620	0.9188
		Inverse	0.9366	0.6750	0.8876	0.9259	0.7105	0.9310
		Squared inverse	0.9251	0.7125	0.8852	0.9331	0.6867	0.9289
	Chebyshev	Equal	0.9308	0.5750	0.8642	0.9048	0.6571	0.9175
		Inverse	0.9337	0.6875	0.8876	0.9284	0.7051	0.9310
		Squared inverse	0.9280	0.6250	0.8712	0.9148	0.6667	0.9209
K = 20	Euclidian	Equal	0.9481	0.5375	0.8712	0.8989	0.7049	0.9229
		Inverse	0.9424	0.6500	0.8876	0.9211	0.7222	0.9316
		Squared inverse	0.9308	0.6500	0.8782	0.9202	0.6842	0.9252
	City block	Equal	0.9452	0.5125	0.8642	0.8937	0.6833	0.9189
		Inverse	0.9424	0.6375	0.8852	0.9185	0.7183	0.9302
		Squared inverse	0.9308	0.6625	0.8806	0.9229	0.6883	0.9256
	Chebyshev	Equal	0.9395	0.5000	0.8571	0.8907	0.6557	0.9144
		Inverse	0.9366	0.6125	0.8759	0.9129	0.6901	0.9245
		Squared inverse	0.9308	0.6250	0.8735	0.9150	0.6757	0.9225

Performance of KNN classifier with experimentally adjusted hyperparameters with the best result marked in bold.

Table 5 shows that the decision tree classifier achieves its highest accuracy of 90.40% using Cross entropy as the split criterion, with a minimum of 4 leaves and a maximum of 5 splits. Alternative settings, such as those using the Gini criterion or higher leaf counts, yield high sensitivity but often lower specificity, leading to reduced accuracy. Therefore, Cross entropy with 4 leaves and 5 splits emerges as the optimal setup for balanced classification on this dataset.

**TABLE 5:** Performance of Decision Tree classifier with different set hyperparameters using 10-fold cross-validation.

Split criterion	Min leaf	Max splits	Sens	Spec	Acc	PPV	NPV	f1
GDI	2	5	0.9164	0.7250	0.8806	0.9353	0.6667	0.9258
		10	0.9251	0.7125	0.8852	0.9331	0.6867	0.9291
		20	0.9107	0.6750	0.8665	0.9240	0.6353	0.9169
		50	0.9049	0.6500	0.8571	0.9181	0.6118	0.9113
	4	5	0.9193	0.7250	0.8829	0.9355	0.6744	0.9273
		10	0.9481	0.6375	0.8899	0.9190	0.7391	0.9333
		20	0.9222	0.6500	0.8712	0.9195	0.6582	0.9208
		50	0.9280	0.6375	0.8735	0.9174	0.6711	0.9223
	8	5	0.9222	0.6250	0.8665	0.9143	0.6494	0.9180
		10	0.9366	0.6125	0.8759	0.9129	0.6901	0.9244
		20	0.9164	0.6375	0.8642	0.9164	0.6375	0.9164
		50	0.9424	0.5875	0.8759	0.9083	0.7015	0.9250
	16	5	0.9164	0.6875	0.8735	0.9271	0.6548	0.9215
		10	0.9049	0.6625	0.8595	0.9208	0.6163	0.9116
		20	0.9078	0.6750	0.8642	0.9238	0.6279	0.9146
		50	0.9337	0.5875	0.8689	0.9076	0.6714	0.9202
**Cross entropy**	2	5	0.9164	0.8375	0.9016	0.9607	0.6979	0.9380
		10	0.9164	0.8125	0.8970	0.9550	0.6915	0.9353
		20	0.9337	0.7125	0.8923	0.9337	0.7125	0.9337
		50	0.9337	0.6500	0.8806	0.9205	0.6933	0.9268
	**4**	**5**	**0.9308**	**0.7875**	**0.9040**	**0.9500**	**0.7241**	**0.9400**
		10	0.9078	0.7875	0.8852	0.9488	0.6632	0.9278
		20	0.9424	0.7375	0.9040	0.9397	0.7468	0.9409
		50	0.9308	0.7125	0.8899	0.9335	0.7037	0.9321
	8	5	0.9020	0.7875	0.8806	0.9485	0.6495	0.9245
		10	0.9280	0.7500	0.8946	0.9415	0.7059	0.9343
		20	0.9308	0.7000	0.8876	0.9308	0.7000	0.9308
		50	0.9251	0.6500	0.8735	0.9198	0.6667	0.9219
	16	5	0.8905	0.7875	0.8712	0.9479	0.6238	0.9179
		10	0.9222	0.6875	0.8782	0.9275	0.6707	0.9248
		20	0.9222	0.7250	0.8852	0.9357	0.6824	0.9289
		50	0.9107	0.7500	0.8806	0.9405	0.6593	0.9251

Performance of Decision Tree classifier with experimentally adjusted hyperparameters with the best result marked in bold.

Another tested method was ANN, see Table 6, which achieved the most accurate results for hidden layers 2, where the size of the first layer is 10 and the size of the second layer is 20 with accuracy of 89.93%. Higher values of layer size and hidden layers already led to worse classification results and were not analyzed further.

**TABLE 6:** Performance of ANN classifier with different set hyperparameters using 10-fold cross-validation.

Hidden layers	Layer size - 1	Layer size - 2	Sens	Spec	Acc	PPV	NPV	f1
**2**	5	5	0.9308	0.5625	0.8618	0.9022	0.6522	0.9163
		10	0.9251	0.7000	0.8829	0.9304	0.6829	0.9277
		20	0.9510	0.6125	0.8876	0.9141	0.7424	0.9322
		50	0.9539	0.5500	0.8782	0.9019	0.7333	0.9277
	**10**	5	0.9395	0.6250	0.8806	0.9157	0.7042	0.9273
		10	0.9337	0.7375	0.8970	0.9391	0.7195	0.9364
		**20**	**0.9337**	**0.7500**	**0.8993**	**0.9419**	**0.7229**	**0.9378**
		50	0.9337	0.6875	0.8876	0.9284	0.7051	0.9309
	20	5	0.9654	0.5625	0.8899	0.9054	0.7895	0.9350
		10	0.9222	0.7125	0.8829	0.9329	0.6786	0.9274
		20	0.9452	0.6875	0.8970	0.9292	0.7432	0.9369
		50	0.9164	0.6500	0.8665	0.9191	0.6420	0.9178
	50	5	0.9049	0.7125	0.8689	0.9318	0.6333	0.9176
		10	0.9510	0.6750	0.8993	0.9270	0.7606	0.9390
		20	0.9366	0.6375	0.8806	0.9181	0.6986	0.9271
		50	0.9337	0.7375	0.8970	0.9391	0.7195	0.9364
1	5	-	0.9539	0.5750	0.8829	0.9068	0.7419	0.9298
	10	-	0.9395	0.6875	0.8923	0.9288	0.7237	0.9340
	20	-	0.9539	0.6375	0.8946	0.9194	0.7612	0.9362
	50	-	0.9280	0.6375	0.8735	0.9174	0.6711	0.9222

Performance of ANN classifier with experimentally adjusted hyperparameters with the best result marked in bold.

Another widely used method is Naive Bayes, with its results presented in Table 7. This method achieved the highest accuracy of 85.25% with the hyperparameter settings ’Prior empirical’ and a ’Distribution Kernel’ of the epanechnikov type. Despite the fact that this method is frequently analyzed, it achieves the worst results in our study compared to other classification methods.

**TABLE 7:** Performance of Naive Bayes classifier with different set hyperparameters using 10-fold cross-validation.

Prior	Distribution	Kernel type	Sens	Spec	Acc	PPV	NPV	f1
Uniform	Normal	-	0.8818	0.6625	0.8407	0.9189	0.5638	0.9000
	Kernel	Normal	0.8156	0.8250	0.8173	0.9529	0.5077	0.8786
		Box	0.8213	0.8000	0.8173	0.9468	0.5079	0.8783
		Epanechnikov	0.8184	0.8125	0.8173	0.9498	0.5078	0.8792
		Triangle	0.8300	0.8125	0.8267	0.9505	0.5242	0.8852
**Empirical**	Normal	-	0.8963	0.6250	0.8454	0.9120	0.5814	0.9039
	**Kernel**	Normal	0.8530	0.7500	0.8337	0.9367	0.5405	0.8930
		Box	0.8818	0.7125	0.8501	0.9301	0.5816	0.9054
		**Epanechnikov**	**0.8761**	**0.7500**	**0.8525**	**0.9383**	**0.5825**	**0.9068**
		Triangle	0.8732	0.7250	0.8454	0.9323	0.5686	0.9019

Performance of Naive Bayes classifier with experimentally adjusted hyperparameters with the best result marked in bold.

In the Random Forest classification method, which operates similarly to decision trees, the highest accuracy was achieved with hyperparameters set to 300 Trees and a Minimum Leaf Size of 1. Increasing the number of trees beyond 500 had no further effect on the overall accuracy of the classifier. It’s worth noting that the best accuracy was obtained with a Minimum Leaf Size of 1, which allows the trees to fit the specific patterns in randomly selected data subsets. However, this can result in trees that are too specialized. Therefore, 10-fold cross-validation was used, with the results shown in Table 8.

**TABLE 8:** Performance of Random Forest classifier with different set hyperparameters using 10-fold cross-validation.

Num. Trees	Min leaf	Sens	Spec	Acc	PPV	NPV	f1
100	1	0.9597	0.6990	0.9018	0.7986	0.9224	0.8720
	2	0.9534	0.6578	0.8948	0.7790	0.9199	0.8574
	3	0.9516	0.6532	0.8945	0.7613	0.9232	0.8478
	4	0.9469	0.6667	0.8901	0.7497	0.9187	0.8395
	5	0.9431	0.6367	0.8874	0.6957	0.9229	0.8016
200	1	0.9539	0.6643	0.9015	0.7576	0.9281	0.8442
	2	0.9432	0.6742	0.8922	0.7230	0.9270	0.8172
	3	0.9478	0.6783	0.8901	0.7427	0.9192	0.8321
	4	0.9512	0.6382	0.8945	0.7464	0.9221	0.8363
	5	0.9501	0.6243	0.8899	0.7479	0.9155	0.8361
**300**	**1**	**0.9529**	**0.6963**	**0.9038**	**0.7800**	**0.9300**	**0.8570**
	2	0.9501	0.6672	0.8971	0.7755	0.9242	0.8535
	3	0.9488	0.6565	0.8969	0.7488	0.9226	0.8396
	4	0.9445	0.6887	0.8970	0.7460	0.9283	0.8357
	5	0.9507	0.5959	0.8946	0.6732	0.9213	0.7889
400	1	0.9515	0.6619	0.8968	0.7464	0.9246	0.8345
	2	0.9452	0.6296	0.8875	0.7216	0.9192	0.8197
	3	0.9514	0.6761	0.8992	0.7713	0.9265	0.8533
	4	0.9423	0.6518	0.8899	0.7334	0.9242	0.8244
	5	0.9503	0.6281	0.8878	0.7767	0.9127	0.8536
500	1	0.9547	0.6574	0.9014	0.7669	0.9273	0.8516
	2	0.9544	0.6498	0.8947	0.7560	0.9204	0.8457
	3	0.9505	0.6351	0.8948	0.7583	0.9245	0.8436
	4	0.9503	0.6719	0.8972	0.7745	0.9236	0.8534
	5	0.9446	0.6204	0.8852	0.7029	0.9166	0.8038

Performance of Random Forest classifier with experimentally adjusted hyperparameters with the best result marked in bold.

Within the Logistic Regression method, the highest detection accuracy of 87.61% was achieved with the hyperparameters: Prior: empirical (class weights are determined based on their frequency in the training data), Regularization: lasso (uses regularization to eliminate irrelevant variables, simplifying the model), and Solver: sparsa (an optimization algorithm efficient for sparse data and large datasets, based on gradient descent). The results of individual logistic regression settings are shown in Table 9. The Table also lists empty values as NaN because these types of hyperparameters settings are not supported.

**TABLE 9:** Performance of Logistic Regression classifier with different set hyperparameters using 10-fold cross-validation.

Prior	Regularization	Solver	Sens	Spec	Acc	PPV	NPV	f1
Uniform	Lasso	sgd	0.8319	0.4072	0.4917	0.3217	0.9674	0.3696
		Asgd	NaN	NaN	NaN	NaN	NaN	NaN
		Dual	NaN	NaN	NaN	NaN	NaN	NaN
		bfgs	NaN	NaN	NaN	NaN	NaN	NaN
		lbfgs	NaN	NaN	NaN	NaN	NaN	NaN
		Sparsa	0.8497	0.8420	0.8501	0.5654	0.9619	0.6800
	Ridge	sgd	0.3528	0.8817	0.4390	0.3613	0.9704	0.3569
		Asgd	NaN	NaN	NaN	NaN	NaN	NaN
		Dual	NaN	NaN	NaN	NaN	NaN	NaN
		bfgs	0.8573	0.8325	0.8524	0.5735	0.9600	0.6853
		lbfgs	0.8591	0.8382	0.8549	0.5873	0.9590	0.6977
		Sparsa	NaN	NaN	NaN	NaN	NaN	NaN
**Empirical**	**Lasso**	sgd	NaN	NaN	NaN	NaN	NaN	NaN
		Asgd	NaN	NaN	NaN	NaN	NaN	NaN
		Dual	NaN	NaN	NaN	NaN	NaN	NaN
		bfgs	NaN	NaN	NaN	NaN	NaN	NaN
		lbfgs	NaN	NaN	NaN	NaN	NaN	NaN
		**Sparsa**	**0.9647**	**0.5325**	**0.8761**	**0.7842**	**0.8941**	**0.8647**
	Ridge	sgd	NaN	NaN	NaN	NaN	NaN	NaN
		Asgd	NaN	NaN	NaN	NaN	NaN	NaN
		Dual	NaN	NaN	NaN	NaN	NaN	NaN
		bfgs	0.9705	0.3868	0.8617	0.7700	0.8727	0.8572
		lbfgs	0.9712	0.3996	0.8640	0.7588	0.8751	0.8530
		Sparsa	NaN	NaN	NaN	NaN	NaN	NaN

Performance of Logistic Regression classifier with experimentally adjusted hyperparameters with the best result marked in bold.

Finally, the last type tested is Ensemble Methods, which are machine learning techniques that combine the outputs of several models (base models) to achieve better performance than any single model alone. Among these methods is Adaptive Boosting, which iteratively improves classification by placing greater emphasis on examples that were misclassified in previous iterations.

In our case, the Tree Learner Template was used, meaning that the base models in the ensemble are Decision Trees. The number of Learning Cycles is set to 200, which determines the number of repetitions during which the models in the ensemble learn and improve their predictions. Higher values for Learning Cycles were not used to prevent potential overfitting of the classifier. With the above hyperparameter settings, the model achieves the highest accuracy of 92.74% compared to the other tested methods. Overall, the results of individual types of Ensemble Methods, see Table 10, achieve better results compared to other methods used in this work based on a different basis.

**TABLE 10:** Performance of Ensemble Method classifiers with different set hyperparameters using 10-fold cross-validation.

Method	Learner template	Learning cycles	Sens	Spec	Acc	PPV	NPV	f1
Adaptive boosting	**Tree**	50	0.9597	0.7250	0.9157	0.9380	0.8056	0.9487
		100	0.9654	0.6750	0.9110	0.9280	0.8182	0.9464
		150	0.9597	0.7125	0.9133	0.9354	0.8028	0.9473
		**200**	**0.9683**	**0.7500**	**0.9274**	**0.9438**	**0.8451**	**0.9558**
	Discriminant	50	0.9481	0.6875	0.8993	0.9294	0.7534	0.9387
		100	0.9452	0.6875	0.8970	0.9292	0.7432	0.9370
		150	0.9481	0.7000	0.9016	0.9320	0.7568	0.9396
		200	0.9452	0.6875	0.8970	0.9292	0.7432	0.9370
Robust boosting	Tree	50	0.9452	0.6875	0.8970	0.9292	0.7432	0.9370
		100	0.9280	0.7000	0.8852	0.9306	0.6914	0.9293
		150	0.9280	0.6750	0.8806	0.9253	0.6835	0.9266
		200	0.9366	0.6625	0.8852	0.9233	0.7067	0.9296
	Discriminant	50	0.9424	0.6250	0.8829	0.9160	0.7143	0.9288
		100	0.9452	0.6750	0.8946	0.9266	0.7397	0.9356
		150	0.9424	0.6750	0.8923	0.9263	0.7297	0.9340
		200	0.9452	0.6875	0.8970	0.9292	0.7432	0.9370
Random undersampling boosting	Tree	50	0.9078	0.8375	0.8946	0.9604	0.6768	0.9334
		100	0.9049	0.7750	0.8806	0.9458	0.6526	0.9249
		150	0.9164	0.7625	0.8876	0.9436	0.6778	0.9296
		200	0.9193	0.7750	0.8923	0.9466	0.6889	0.9319
	Discriminant	50	0.9049	0.7750	0.8806	0.9458	0.6526	0.9249
		100	0.8991	0.7875	0.8782	0.9483	0.6429	0.9228
		150	0.9107	0.7875	0.8876	0.9489	0.6702	0.9286
		200	0.8963	0.7875	0.8759	0.9482	0.6364	0.9217
Adaptive logistic regression	Tree	50	0.9539	0.7000	0.9063	0.9324	0.7778	0.9429
		100	0.9597	0.7000	0.9110	0.9328	0.8000	0.9459
		150	0.9654	0.6625	0.9087	0.9254	0.8154	0.9451
		200	0.9597	0.6625	0.9040	0.9250	0.7910	0.9420
Gentle adaptive boosting	Tree	50	0.9654	0.7250	0.9204	0.9384	0.8286	0.9517
		100	0.9625	0.7000	0.9133	0.9330	0.8116	0.9474
		150	0.9597	0.7250	0.9157	0.9380	0.8056	0.9473
		200	0.9625	0.7500	0.9227	0.9435	0.8219	0.9526

Performance of Ensemble Method classifier with experimentally adjusted hyperparameters with the best result marked in bold.

The best results for each machine learning method are highlighted in bold in the respective tables. Of all the tested individual classification methods with experimentally set hyperparameters, the Ensemble Methods achieved the highest classification accuracy with an accuracy of 92.74%, a sensitivity of 96.83% and a specificity of 75.00%. Due to the class imbalance (80 H C vs. 347 MI) and the chosen ROC operating point, the specificity metric is highly sensitive to even a small number of false positives from the HC group. Among other things, the parameters PPV and NPV are also presented here, where PPV tells us the proportion of subjects with a positive test result who truly have the outcome of interest, while NPV reflects the proportion of subjects with a negative test result who truly do not have the outcome of interest.

**FIGURE 6:**
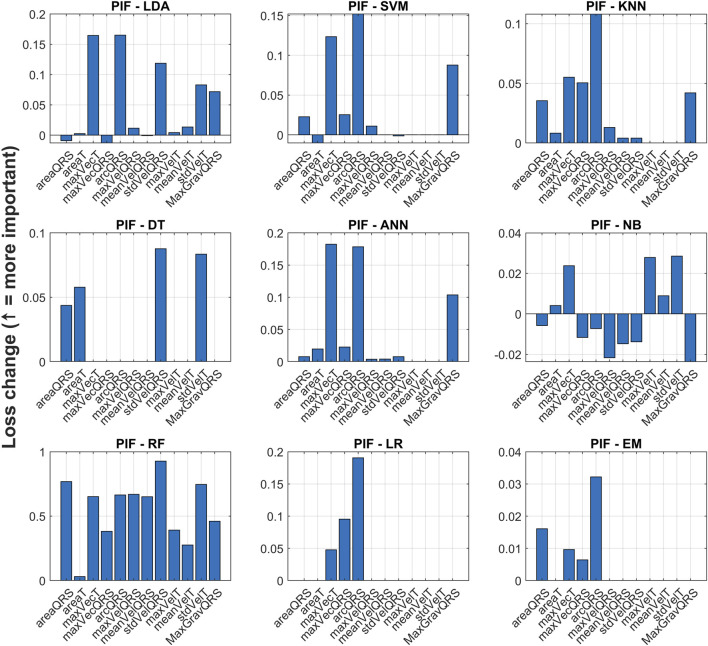
Permutation Importance of Features across all nine classifiers. The y-axis represents the increase in classification loss when a given feature is randomly permuted–higher values indicate greater importance.

To better understand which features contributed most to model performance, permutation importance of features (PIF) was computed for all nine base classifiers, see Figure 6. This method estimates the importance of each feature by measuring the increase in classification error when the feature values are randomly shuffled. The higher the increase in loss, the more critical the feature is to the model’s predictions. Positive PIF values indicate that shuffling the feature increases the model’s error, suggesting that the feature is important for prediction. In contrast, negative PIF values imply that randomizing the feature slightly improves the model performance, which may indicate redundancy or noise. As shown in Figure 6, certain features consistently showed higher importance across multiple classifiers–such as maxVelT, arcQRS, and maxVecQRS–suggesting their robust discriminative power for myocardial infarction detection. Notably, logistic regression, decision tree, and artificial neural network placed strong emphasis on a small subset of features, while methods like Random Forest and KNN demonstrated a more balanced distribution of feature importance.

Finally, the stacking ensemble learning method was employed for classification, utilizing decision-level fusion by combining predictions from multiple base models to enhance classification performance. The input for logistic regression as a meta-classifier consisted of the best-performing trained models (basic models from Table 11), ensuring that only the most informative classifiers contributed to the final prediction. To avoid data leakage, OOF predictions from the base models were used as inputs. This means that each base classifier generated predictions only for samples that were not included in its training fold. The strong performance of the stacking approach can be attributed to the behavior of the individual base classifiers, which shows diverse error patterns across different patient groups. This diversity allowed the meta-classifier to effectively learn how to weigh individual model outputs. In particular, methods such as SVM, DT and ANN contributed different decision boundaries and sensitivities to feature variations. These different boundaries can together improve the final prediction. The results demonstrate high sensitivity of 97.70%, specificity of 86.25%, accuracy of 95.55%, PPV of 96.86%, NPV of 89.61%, and f1-score of 97.27%. These results confirm the effectiveness of the fusion-based approach, where decision-level integration of multiple classifiers improved robustness. This analysis also supports the conclusion that the success of a layering model lies not only in the combination of strong individual classifiers, but also in exploiting their diversity to mitigate individual weaknesses.

**TABLE 11:** Summary of the performance parameters of individual classification methods that achieved the most accurate results.

Classification method	Sensitivity (%)	Specificity (%)	Accuracy (%)	PPV (%)	NPV (%)	f1-score (%)
KNN	93.66	73.75	89.93	93.93	72.84	93.79
SVM	95.10	73.75	91.10	94.02	77.63	94.55
DT	93.08	78.75	90.40	95.00	72.41	94.03
DA	95.10	70.00	90.40	93.22	76.71	94.15
ANN	93.37	75.00	89.93	94.19	72.29	93.78
NB	87.61	75.00	85.25	93.83	58.25	90.61
RF	95.29	69.63	90.38	93.00	78.00	94.13
LR	96.47	53.25	87.61	89.41	78.42	92.81
**EM**	**96.83**	**75.00**	**92.74**	**94.38**	**84.51**	**95.59**
**STACKING**	**97.70**	**86.25**	**95.55**	**96.86**	**89.61**	**97.27**

Performance of basic models with the EM method selected, which achieved the best results individually, and the STACKING method as a combination of all basic models.

**FIGURE 7:**
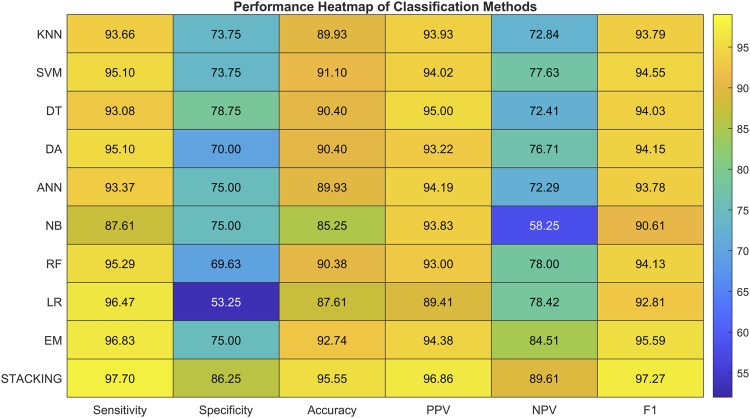
Heatmap visualization of classification performance metrics for all evaluated methods. The stacking and ensemble models achieved consistently higher scores across all metrics.

Figure 7 shows a heatmap comparing the performance metrics of all classifiers. It highlights the superior results of the stacking method, followed by strong performance from SVM, DA, and RF, while NB and LR performed worse in specificity and NPV. This graphical representation helps illustrate the overall superiority of ensemble-based methods compared to single classifiers.

**FIGURE 8:**
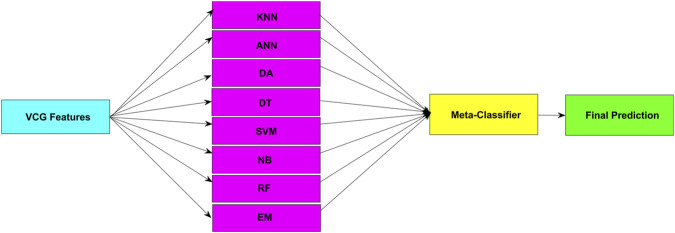
Block diagram of the Stacking method principle combining M-L models into a meta-classifier.

Figure 8 illustrates the principle of the Stacking method, which integrates multiple M-L models into a single meta-classifier to enhance detection accuracy. This meta-classifier receives the outputs of the base M-L models, providing it with additional information for better decision-making. In cases of misclassifications by the base M-L models, the meta-classifier learns to recognize patterns in these errors and optimizes the final prediction.

**FIGURE 9:**
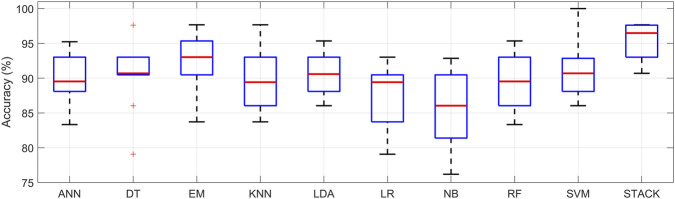
Output accuracy variability for individual M-L methods using 10-fold cross-validation.

The aforementioned M-L methods were analyzed using 10-fold cross-validation, where each iteration produces a specific accuracy value for detection. This variability is shown in Figure 9 for the M-L methods that achieved the best results, as listed in Table 11. It can be observed that the accuracy variability in the Stacking method is lower compared to individual M-L models, highlighting the robustness of this approach.

Table 11 shows summarization of the performance parameters for the classification methods with set hyperparameters that achieved the most accurate results. Correspondingly, Table 12 presents the confusion matrix for these methods, as detailed in Table 7. From the confusion matrices can be observed that the base classifiers made different types of errors. For example, KNN and DT tended to misclassify borderline HC as MI, while SVM and DA performed better in these cases but occasionally failed to detect atypical MI. ANN showed higher sensitivity but slightly lower specificity, reflecting its tendency to overestimate positive cases. LR achieved high sensitivity, but also achieved a higher false positive rate. These variations in error patterns indicate a high degree of model diversity, which the stacking ensemble effectively exploited.

**TABLE 12:** The confusion matrices for the analyzed machine learning methods that achieved the most accurate results.

KNN			SVM			DT			DA			ANN		
	MI	HC		MI	HC		MI	HC		MI	HC		MI	HC
MI	325	22	MI	330	17	MI	323	24	MI	330	17	MI	324	23
HC	21	59	HC	21	59	HC	17	63	HC	24	56	HC	20	60

NB			RF			LR			EM			STACKING		
	MI	HC		MI	HC		MI	HC		MI	HC		MI	HC
MI	304	43	MI	331	16	MI	335	12	MI	336	11	MI	339	8
HC	20	60	HC	24	56	HC	37	43	HC	20	60	HC	11	69

**FIGURE 10:**
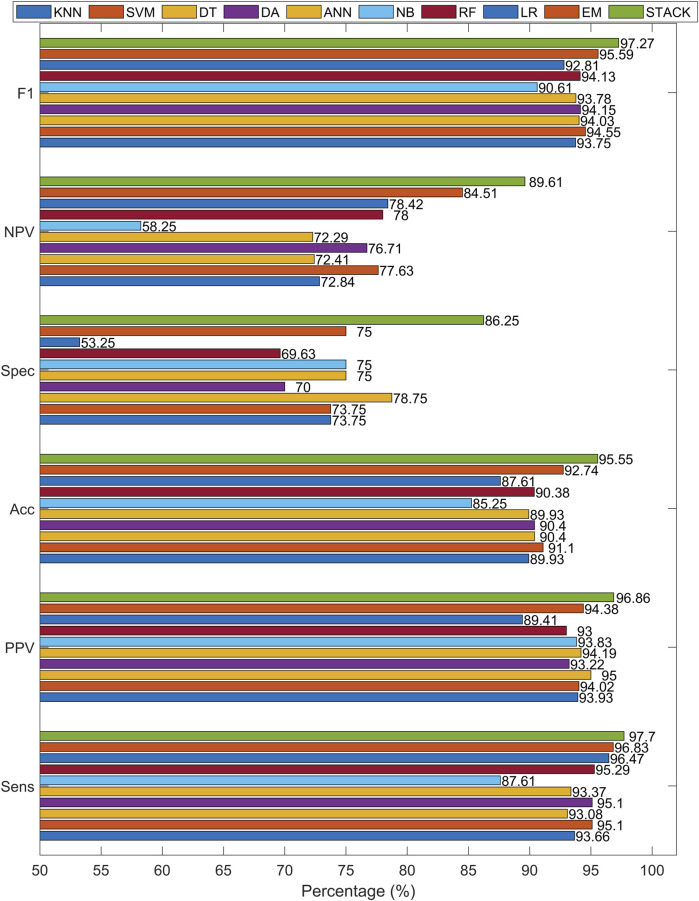
Comparison of values of sensitivity, specificity, accuracy, PPV, NPV and f1-score for individual analyzed machine learning methods with set hyperparameters that achieved the most accurate results.

**FIGURE 11:**
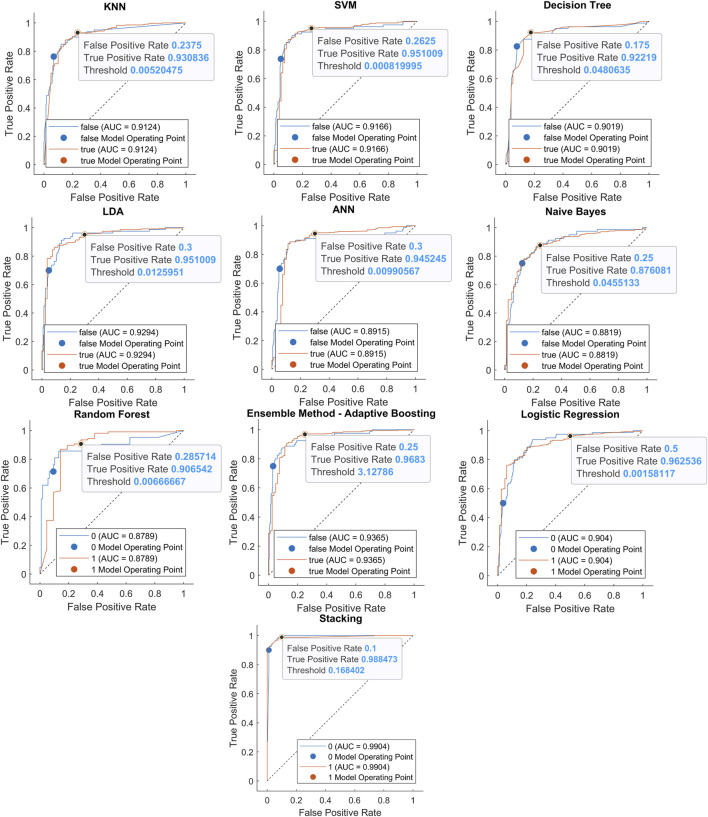
ROC with indication of the true and false operating point of classifiers: KNN with K = 10, distance type City Block, distance weight Squared Inverse; SVM with kernel type Polynomial and Order 3, Scale 1; DT with split criterion Cross entropy, Min. leaf 4, Max. splits 5; DA with discriminant type Linear; ANN with 2 hidden layers, layer 1 size 10 and layer 2 size 20, which achieve the highest accuracy; NB with Prior empirical, Distribution Kernel type of epanechnikov; RF with Number of trees 300, Min leaf size 1; LR with Prior empirical, Regularization lasso, Solver sparsa; EM as Adaptive Boosting, Learner template Tree, learning cycles 200; Stacking ensemble learning combining M-L method models.

Another way to illustrate the results is shown in Figure 10, which corresponds to the data presented in Table 12. From these visuals, it is evident that the Ensemble Method delivers the highest accuracy among all the tested methods. Given the class imbalance in the dataset (347 MI vs. 80 healthy), we also show ROC curves and AUC (area under curve) as performance metrics. Unlike accuracy, AUC is less sensitive to class imbalance and provides a more reliable evaluation of model performance across thresholds, see Figure 11, with the marked cut-point of the ROC curve (Habibzadeh et al., 2016). This cut-point is obtained from the true ROC curve representing the MIs. ROC curves describe the quality of a binary classifier depending on the setting of its classification threshold. Similarly, the cut-point can be obtained from the false ROC curve, which in this case would be the mirror values of the true curve. Table 13 presents the AUC values for the analyzed classifiers that achieved the highest accuracy. The stacking method reached the highest AUC value of 99.04%, confirming its ability to distinguish between classes. Although techniques such as SMOTE or cost-sensitive learning could be applied to mitigate class imbalance, we opted to evaluate model performance without resampling in order to preserve the natural class distribution. Nonetheless, we highlight this as a limitation and propose exploring such methods in future work.

**TABLE 13:** AUC values for the analyzed M-L methods according to the Table 11.

M-L Method	KNN	SVM	DT	LDA	ANN	NB	RF	EM	LR	Stack
AUC (%)	91.24	91.66	90.19	92.94	89.15	88.19	87.89	93.65	90.40	**99.04**

## 4 Discussion

VCG features are a suitable parameter that can capture morphological changes in physiological and pathological recordings. The advantage of the used domain knowledge VCG features is the capture of morphological changes in the QRS and T loop, which is changed in the presence of pathology. We analyzed a total of 12 VCG features that were extracted from 80 physiological and 347 pathological recordings from PTB Physionet database. Each of these features was tested by the M-W statistical test to verify its predictive value. Since all features had a p-value less than 0.05, see Table 1, all features were fed into the classification methods.

Various methods have been investigated for classification, including KNN, SVM, ANN, DA, DT, NB, RF, LR, EM, and Stacking Ensemble Learning. Each of these classifiers was trained using 10-fold cross-validation. To maximize the information obtained, each of these classifiers was analyzed with experimentally adjusted hyperparameters. The performance of individual classifiers was investigated using various parameters, including Sensitivity, Specificity, Accuracy, Positive Predictive Value, and Negative Predictive Value. It can be observed from Table 11 that the most accurate classification results were achieved by the Stacking Ensemble Learning method, which combined the predictions of multiple base classifiers and used logistic regression as a meta-classifier. This approach yielded an accuracy of 95.55%, a sensitivity of 97.70%, a specificity of 86.25%, a positive predictive value of 96.86%, a negative predictive value of 89.61% and f1-score of 97.27%. To ensure the robustness of the used classifiers and to prevent overfitting, the 10-fold cross-validation method was applied, where the analyzed data was divided into 10 subsets with samples having approximately equal distribution in each group.

### 4.1 Comparison with existing studies

The following paragraphs compare the achieved results with existing studies. The focus is primarily on a similar problem, namely the extraction of VCG features and machine learning methods for the possibility of automatic pathology detection.

Among recent studies focused on MI detection, the authors in Correa et al. (2013b) analyzed a total of seven QRS features and their classification ability, achieving a sensitivity of 88.5% and a specificity of 92.1%. The authors further expanded their analysis in Correa et al. (2013a), where they extracted 4 features analyzing the QRS loop and 3 features analyzing the T loop for 51 ischemic and 52 healthy subjects. Using the LDA classifier, they achieved a sensitivity of 95.4% and a specificity of 95.2%. The authors also devoted their attention to the classification of anterior and inferior infarctions in Correa et al. (2016), where they achieved an accuracy of 89.8% using nine features and the LDA classifier. The spatial velocity dynamics of the QRS loop VCG of patients with AMI was also analyzed by Ghosal et al. (2024). Using quasi-orthogonal leads I, aVF, and V2, they constructed the VCG and examined spatial velocity, spatial distance and spatial magnitude. The results showed decreased spatial velocity and spatial distance values in patients with AMI. Another important analysis of VCG features was performed by the authors in De la Garza Salazar and Egenriether (2024), where they analyzed 315 VCG parameters from the P, QRS and T loops using derived VCG via the Kors transformation (Kors et al., 1990). They identified significant associations between VCG features and patient characteristics, such as age, sex, BMI, hypertension, and echocardiographic findings. The results highlight the potential of VCG analysis in cardiovascular disease assessment.

Dehnavi et al. (2011) compared the success of MI detection from ECG and VCG recordings. For this purpose, they extracted 22 features from 60 ischemic and 10 healthy recordings and used a neural network for classification. They observed a significant improvement in the processing of VCG data, achieving an accuracy of 86%, compared to 73% for ECG. Zhao et al. (2022) used a combination of features extracted from ECG and VCG and achieved accuracy of 90.3%, sensitivity of 90.3% and specificity of 90.5% using SVM classifier. Additionally, Dima et al. (2013) used 25 features extracted from 158 MI and 52 H C recordings to detect MI. For classification, they employed an SVM classifier, which resulted in an accuracy of 89.22%, sensitivity of 76%, and specificity of 87.5%. SVM was also used as a classification method by the authors in Panagiotou et al. (2013); Khan and Pachori (2021a). In Panagiotou et al. (2013), the authors extracted 27 morphological features from 158 pathological and 102 healthy records, achieving an accuracy of 82.36%, sensitivity of 84.31%, and specificity of 77.36%. In contrast, the authors in Khan and Pachori (2021a) used 12 features based on the frequency components of the signal, achieving an accuracy of 95.52%, sensitivity of 91.08%, and specificity of 97.45%. Another use of features based on the wavelet transform of the signal was used by the authors in Keshtkar et al. (2013). The authors proposed the evaluation of the wavelet coefficients obtained over the averaged ECG signal. From the obtained set of parameters and using a neural network, they achieved an accuracy of 89.5%, a sensitivity of 93% and a specificity of 86%. Tripathy and Dandapat (2017) also obtained VCG features using wavelet transformation. In their analysis, they employed the relevance vector machine method, using 100 MI and 50 H C records, which resulted in a sensitivity of 98.40% and specificity of 98.66%. Furthermore, the authors in Zhang J. et al. (2022) detected MI localization with a detection success rate exceeding 99% using the decomposition of the detected individual beats using the wavelet transform. However, these high detection success rates in the above-mentioned works dealing with signal decomposition using wavelet transformation are limited by the high computational complexity and the need for a large operating memory, as stated for example in Zhang J. et al. (2022).

The concept of ensemble learning was also applied by the authors in Sun et al. (2022), who extracted features from transformed VCGs based on the spectral fitting exponent, Lyapunov exponent, and Lempel-Ziv complexity. These features were processed through an ensemble learning algorithm based on bagging, combining various classifiers using weighted voting. The proposed algorithm achieved a detection accuracy of 91.11%, sensitivity of 90.49%, and specificity of 92.88% on the PTB database. Hafshejani et al. (2021) applied the Classification and Regression Tree (CART) method on a balanced dataset consisting of 80 MI and 80 H C records. Using octant theory, they extracted a total of 48 features. With the dataset split into 90% for training and 10% for testing, the authors achieved an accuracy of 98.1%, sensitivity of 98.8%, and specificity of 97.5%. Karisik and Baumert (2019) extracted VCG features based on the morphological properties of the QRS and T loops, and using a long short-term memory network, they achieved MI detection accuracy of 89.1%, with a sensitivity of 89.1% and a specificity of 90%. In their analysis, they used 78 MI and 69 H C records from the PTB database. The authors in Aranda Hernandez et al. (2023) also applied ensemble methods for detecting various types of MI, focusing on the automatic localization of MI using four different M-L methods with 98 VCG features as input data. They report that Lasso or ensemble models can achieve better detection results in situations with limited data, compared to more complex models based on deep learning approaches.

Attention was also paid to comparing different types of classifiers using VCG features analyzing signal decompositions using wavelet variation Sharma and Sunkaria (2018); Khan and Pachori (2021b), where in Sharma and Sunkaria (2018) authors analyzed two classification methods (SVM and KNN) to see which one would perform better. From the dataset of features based on signal decomposition, they achieved a higher accuracy of detection with the SVM method, which achieved very good results: sensitivity 99.35%, specificity 98.29% and accuracy 98.41%. However, it should be mentioned that the authors in Sharma and Sunkaria (2018) used 30 pathological and 52 healthy subjects. Gragnaniello et al. (2024) compared two methods for MI detection based on M-L and deep learning (D-L) techniques from a proposed single microcontroller-based system. They used 4-s recordings from the PTB database for analysis and achieved detection accuracy of 89.40% for M-L and 94.76% for D-L.

We also present a comparison with relevant publications analyzing a similar problem is shown in Table 14, where attention is mainly paid to the number of extracted features, the number of records used and the M-L method used.

**TABLE 14:** A summary of selected relevant publications related to the topic of this work analyzing VCG features using machine learning methods for myocardial infarction detection.

Authors	Year	M-L Method	Num. Of records	Num. Of features	Performance (%)
Dehnavi et al. (2011)	2011	Forward neural networks	60 ischemic; 10 H C	22	Sens: 70.00 Spec: 86.00
Correa et al. (2013b)	2013	LDA	82 MI; 52 H C	8	Sens: 88.50 Spec: 92.10
Keshtkar et al. (2013)	2013	Probabilistic neural networks	50 MI; 50 H C	25	Sens: 93.00 Spec: 86.00
Dima et al. (2013)	2013	SVM	158 MI; 52 H C	25	Sens: 76.00 Spec: 87.50
Panagiotou et al. (2013)	2013	SVM	112 MI; 80 H C	8	Sens: 84.31 Spec: 77.36
Goernig et al. (2015)	2015	Discrimination analysis	59 MI; 55 H C	8	Sens: 71.70 Spec: 88.70
Correa et al. (2016)	2016	LDA	95 MI; 52 H C	9	Acc: 89.80
Tripathy and Dandapat (2017)	2017	Relevance vector machine	100 MI; 50 H C	15	Sens: 98.40 Spec: 98.66
Hernandez et al. (2018)	2018	The gradient boosting	98 ischemic before (control) and during PTCA	7 out of 328	Sens: 89.60 Spec: 82.70
Diker et al. (2018)	2018	SVM	148 MI; 52 H C	23	Acc: 87.80
Sharma and Sunkaria (2018)	2018	SVM and KNN	368 MI; 80 H C	10	Sens: 79.01 Spec: 79.26
Prabhakararao and Dandapat (2019)	2019	SVM	4 PMI; 80 H C	12	Acc: 96.69 Sens: 80.00 Spec: 98.40
Prabhakararao and Dandapat (2020)	2020	SVM	13 PMI; 80 H C	12	Acc: 96.94
Hafshejani et al. (2021)	2021	Classification and regression tree	80 MI; 80 H C	48	Sens: 98.80 Spec: 97.50
Khan and Pachori (2021a)	2021	SVM	13 PMI; 50 H C	12	Sens: 91.08 Spec: 97.45
Zhao et al. (2022)	2022	SVM	148 ischemic; 52 H C	3	Acc: 90.30
Sun et al. (2022)	2022	Heterogeneous ensemble	383 myocardial ischemia; 116 H C	6	Acc: 91.11 Sens: 90.49 Spec: 92.88
Aranda Hernandez et al. (2023)	2023	Lasso ensemble method	148 MI; 52 H C	98	AUC: 81.45 Sens: 75.80 Spec: 87.80
Zeng and Yuan (2023)	2023	Dynamical estimators	148 MI; 52 H C	> 20	Acc: 98.20 Sens: 98.37 Spec: 97.44
**This work**	**2025**	Stacking ensemble learning	**347 MI; 80 H C**	**12**	**Acc: 95.55** **Sens: 97.70** **Spec: 86.25**

### 4.2 Advantages and limitations of proposed study

It is worth mentioning that the authors in Khan and Pachori (2021b) analyzed three different classifiers for posterior MI (PMI) detection by using derived VCG from a 12 lead ECG, whereas, in the present study, attention is paid to the possibility of MI detection from directly measured VCG records. In contrast, in the present study, we extracted 12 features that analyze the morphological properties of cardiac revolution caused by infarct conditions, while in Khan and Pachori (2021b) authors used features based on signal decomposition using a specialized wavelet transform. In this work, VCG features can be considered as mathematical operations applied to VCG signals that do not require high computational complexity and can be applied to any recording. Compared to other publications, this work ranks among the publications using a lower number of features, which may affect the subsequent computational complexity. These features were analyzed to verify their informative value and their relevance was confirmed using statistical analysis.

The present work also addresses the issue of utilizing a larger or comparable number of patient records compared to the previous studies, as indicated in Table 14 and the chapter dealing with comparison with existing studies. It is evident from the table that different authors use varying numbers of records from the same database for their analyses. This inconsistency can be misleading, as some studies do not specify which subset of data was used. We are also aware of the imbalance between the MI and HC groups, which is subsequently reflected in the specificity values in our study. Achieving consistently high specificity is particularly challenging when working with a relatively small number of HC. Even a few misclassified cases can influence this metric. Nevertheless, the obtained values correspond to the selected operating point on the ROC curve, which reflects the trade-off between sensitivity and specificity and was chosen to favor higher sensitivity due to its greater clinical importance in MI detection. This choice inevitably leads to a slight decrease in specificity, which becomes more apparent given the small HC cohort. Furthermore, due to the imbalance between the analyzed groups (MI and HC), additional metrics such as NPV, PPV, and f1-score were used, which is more appropriate in this case. Another challenge in the literature is the unavailability of data used by some authors who do not rely on publicly accessible databases. The limited availability of VCG data remains a barrier to comprehensive analyses of VCG recordings. Future research may focus on developing a modern database of ECG and VCG records to address this limitation.

Furthermore, this work examines commonly used machine M-L for detecting various heart diseases based on ECG and VCG recordings. A review of methods applied in the last 5 years was conducted. The analyzed M-L methods were experimentally optimized to determine their best performance through a comprehensive comparative analysis. The results of MI detection using M-L methods, combined in a Stacking ensemble model, achieve better results compared to the state-of-the-art, particularly in the analysis of morphological features in both the time and frequency domains. To the best of our knowledge, the used combination of features based on the morphological properties of cardiac revolution with verification of their predictive value using a statistical test and the detailed analysis of classifiers with experimentally set hyperparameters with subsequent creation of a Stacking ensemble learning model with high detection success rate has been used for the first time on directly measured VCG recordings in order to find a suitable methodology for detecting MI and a subsequent option to support diagnostics in clinical practice. This work contributes to the existing literature by demonstrating the benefits of MI detection based on a detailed analysis of M-L methods using morphological VCG features, followed by the design of a Stacking model of M-L methods. The results achieved have the potential to enhance clinical diagnostics.

Furthermore, this work offers additional potential directions for addressing the given issue, such as extracting additional VCG features that could enhance the informative value within classification, or applying the described methodology to transformed VCG recordings derived from 12-lead ECG, which is the most commonly measured in clinical practice. These derived VCGs could provide complementary information to existing ECG-based diagnostic systems and support clinicians in their decision-making. The 12-lead ECG, routinely used in clinical examinations, can be mathematically transformed into VCG leads using established transformation methods. Such reconstructed VCG recordings expand the scope of cardiac electrical activity analysis and can serve as a valuable supplement to conventional ECG interpretation. From these derived VCG leads, the same set of VCG features can be extracted, and the described classification approach using both standard machine learning and stacking techniques can be applied. In this way, the proposed method could be implemented on already acquired ECG data for diagnostic verification or integrated into real-time clinical analysis, thereby contributing to the improvement of the necessary treatment.

## 5 Conclusion

Early diagnosis and detection of myocardial infarction can help physicians provide timely and effective treatment, potentially saving the lives of at-risk patients. In this study, a methodology for processing of vectorcardiographic records for the automated detection of MI records was proposed, based on the analysis of extracted VCG features analyzing the morphological properties of the QRS and T loop. Among 210 different M-L settings, the highest accuracy was achieved using the Stacking Ensemble Learning method, which combines multiple base models and employs logistic regression as a meta-classifier. This approach yielded high accuracy of 95.55%, sensitivity of 97.70%, specificity of 86.25%, positive predictive value of 96.86%, negative predictive value of 89.61%, and f1-score of 97.27%. These results confirmed the usefulness of the VCG method and can aid physicians in decision-making regarding subsequent treatment plans.

## Data Availability

Publicly available datasets were analyzed in this study. This data can be found here: https://physionet.org/content/ptbdb/1.0.0/.
