# Supplementary figures and images for: Enhancing myocardial infarction detection with vectorcardiography: fusion-based comparative analysis of machine learning methods
Source: Front Physiol. 2026 Jan 5;16:1683956. doi: 10.3389/fphys.2025.1683956 (PMC12813126; doi:10.3389/fphys.2025.1683956)

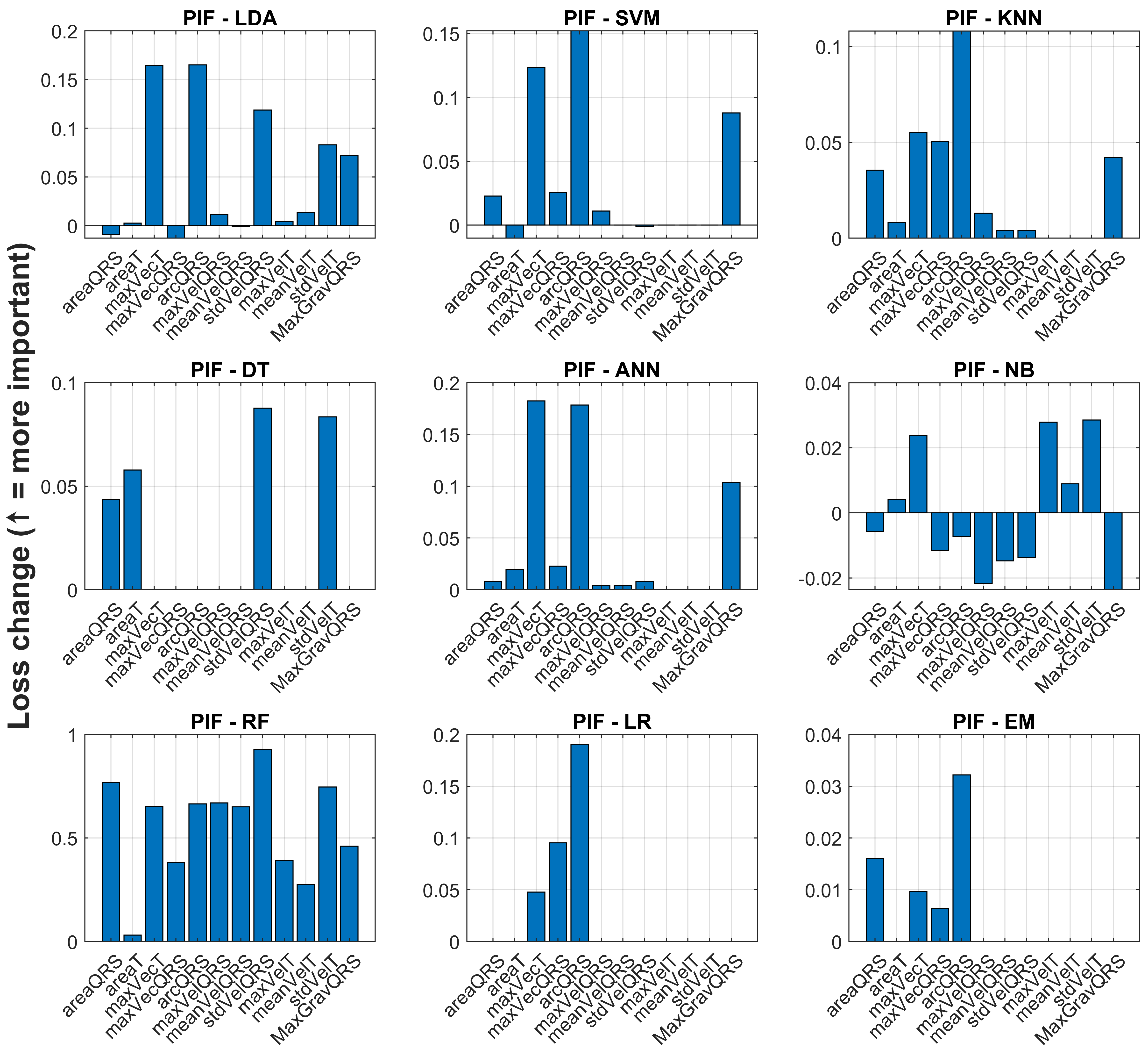

Supplement: Supplementary file 1 [file Image11.PNG]

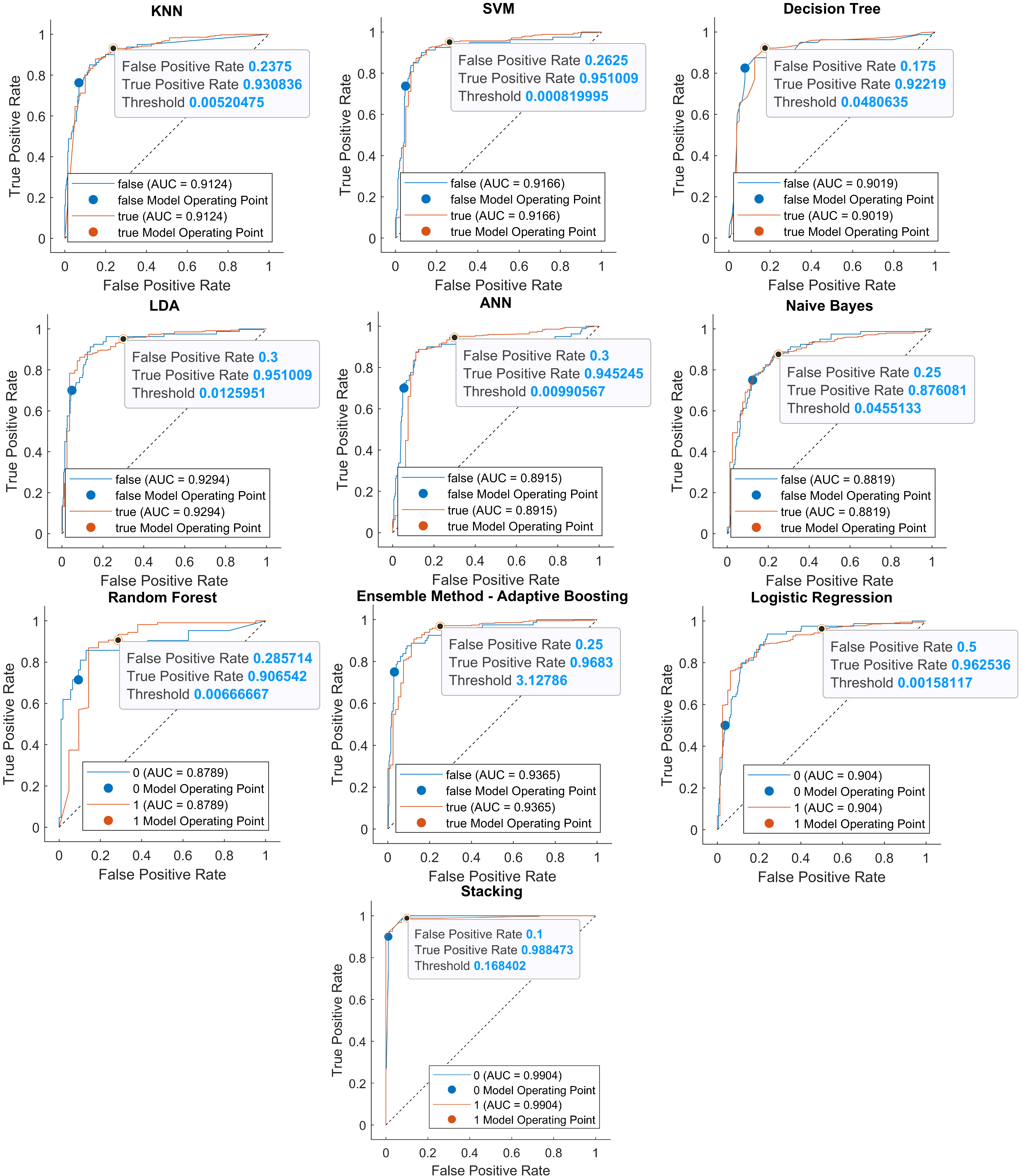

Supplement: Supplementary file 2 [file Image12.PNG]

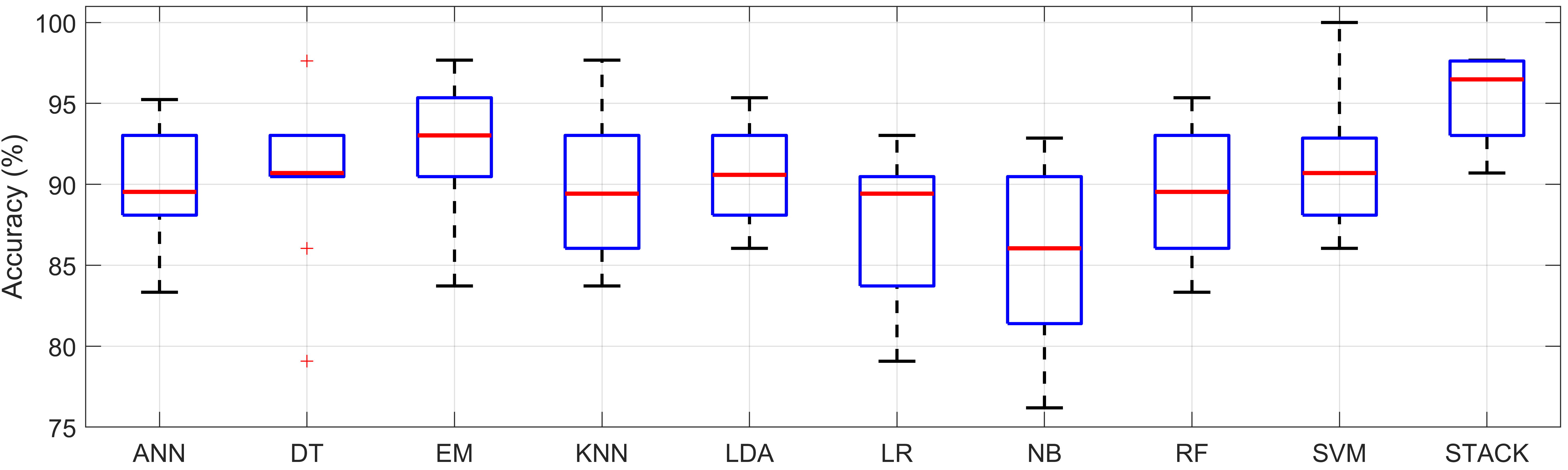

Supplement: Supplementary file 3 [file Image5.PNG]

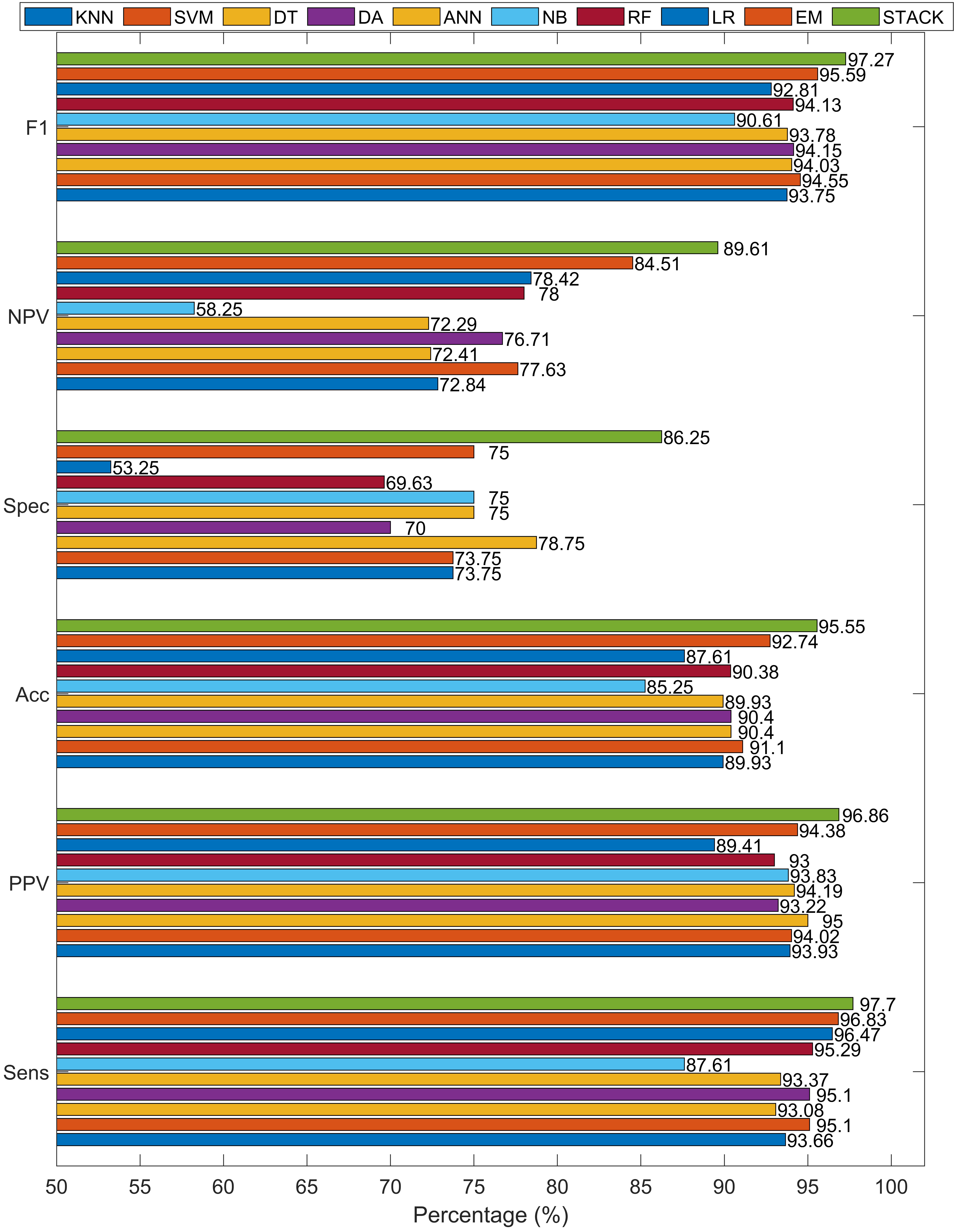

Supplement: Supplementary file 4 [file Image4.PNG]

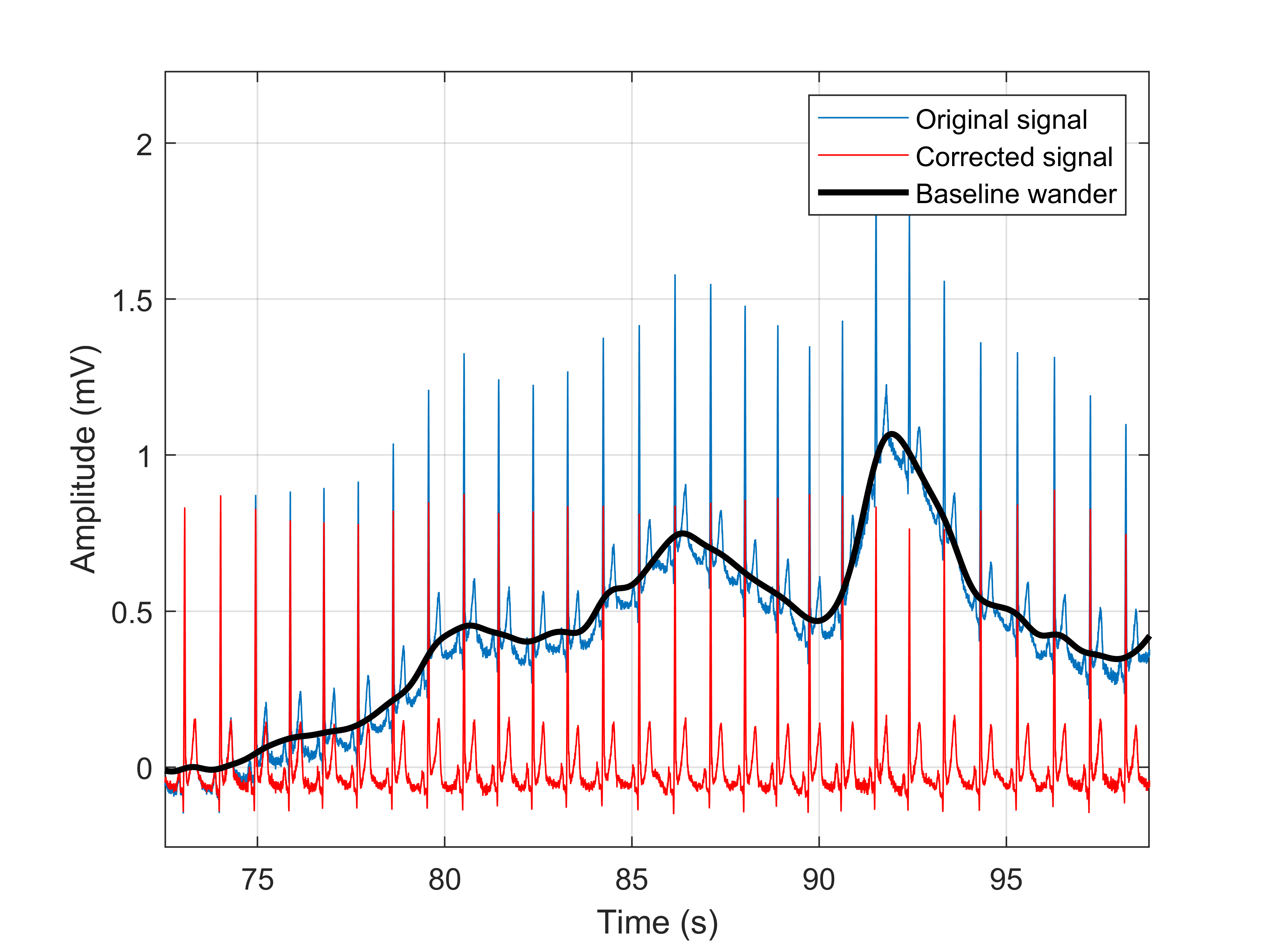

Supplement: Supplementary file 6 [file Image7.PNG]

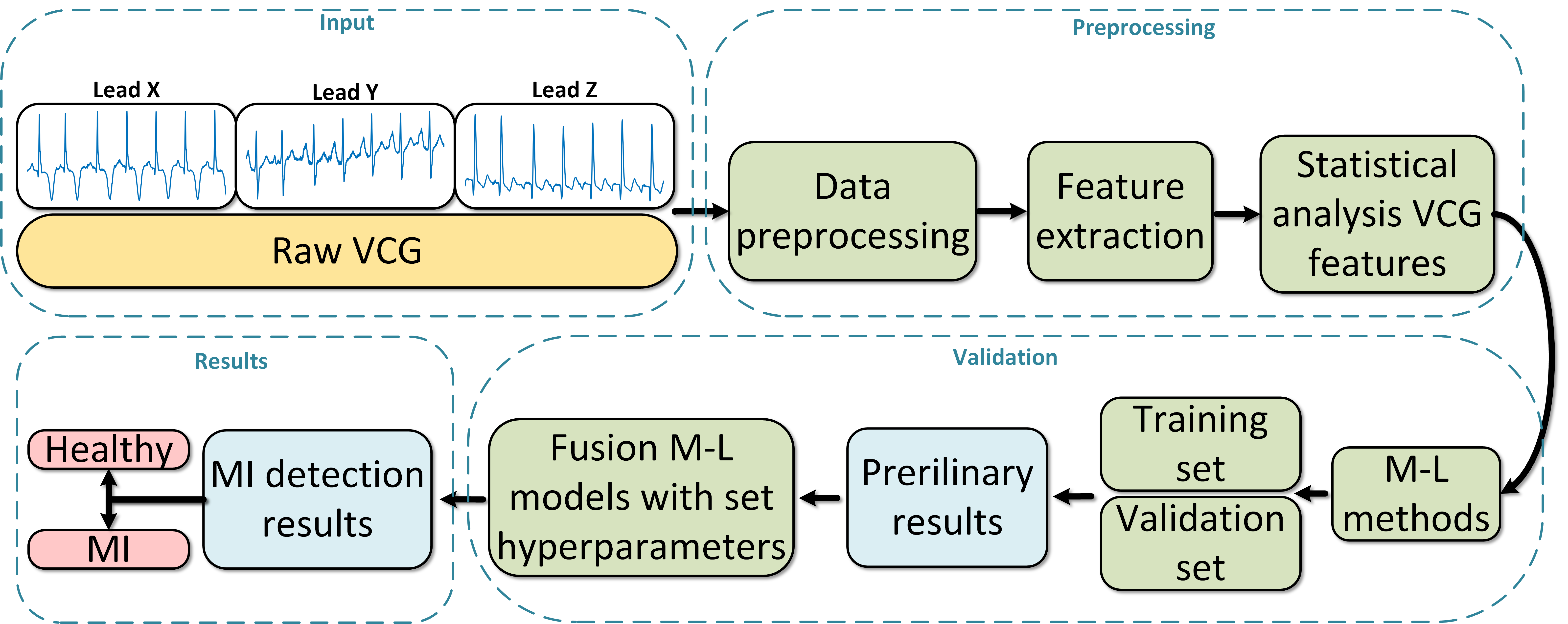

Supplement: Supplementary file 7 [file Image2.PNG]

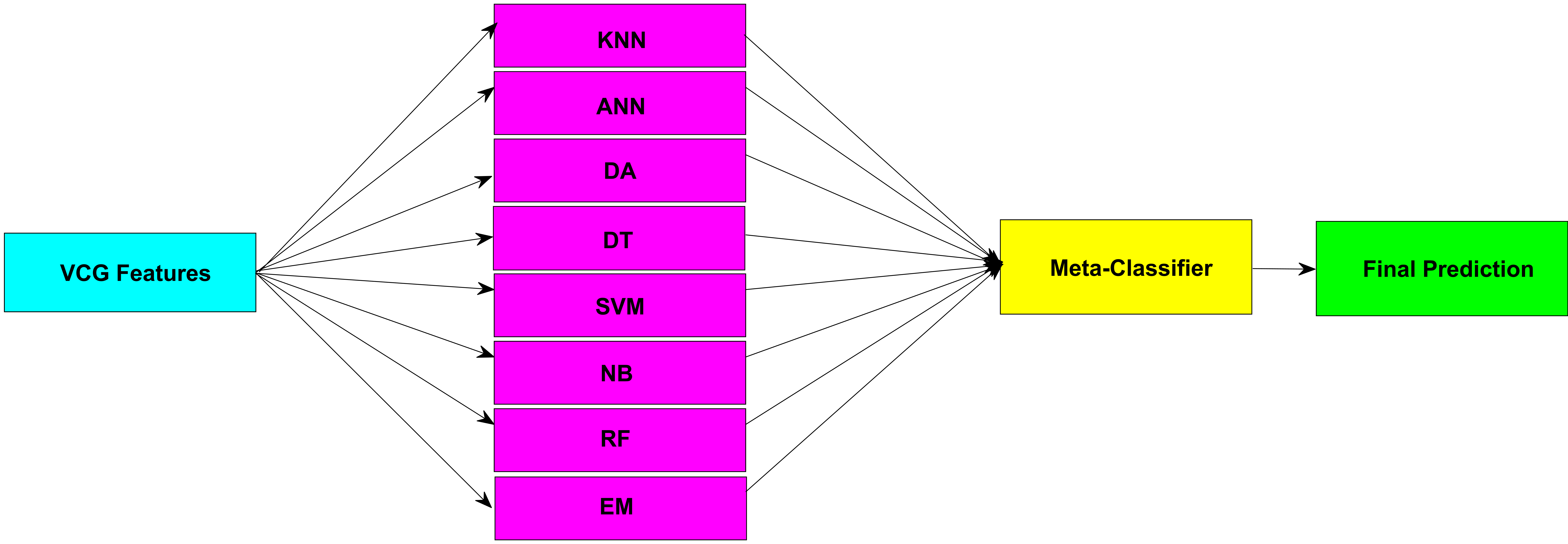

Supplement: Supplementary file 8 [file Image1.PNG]

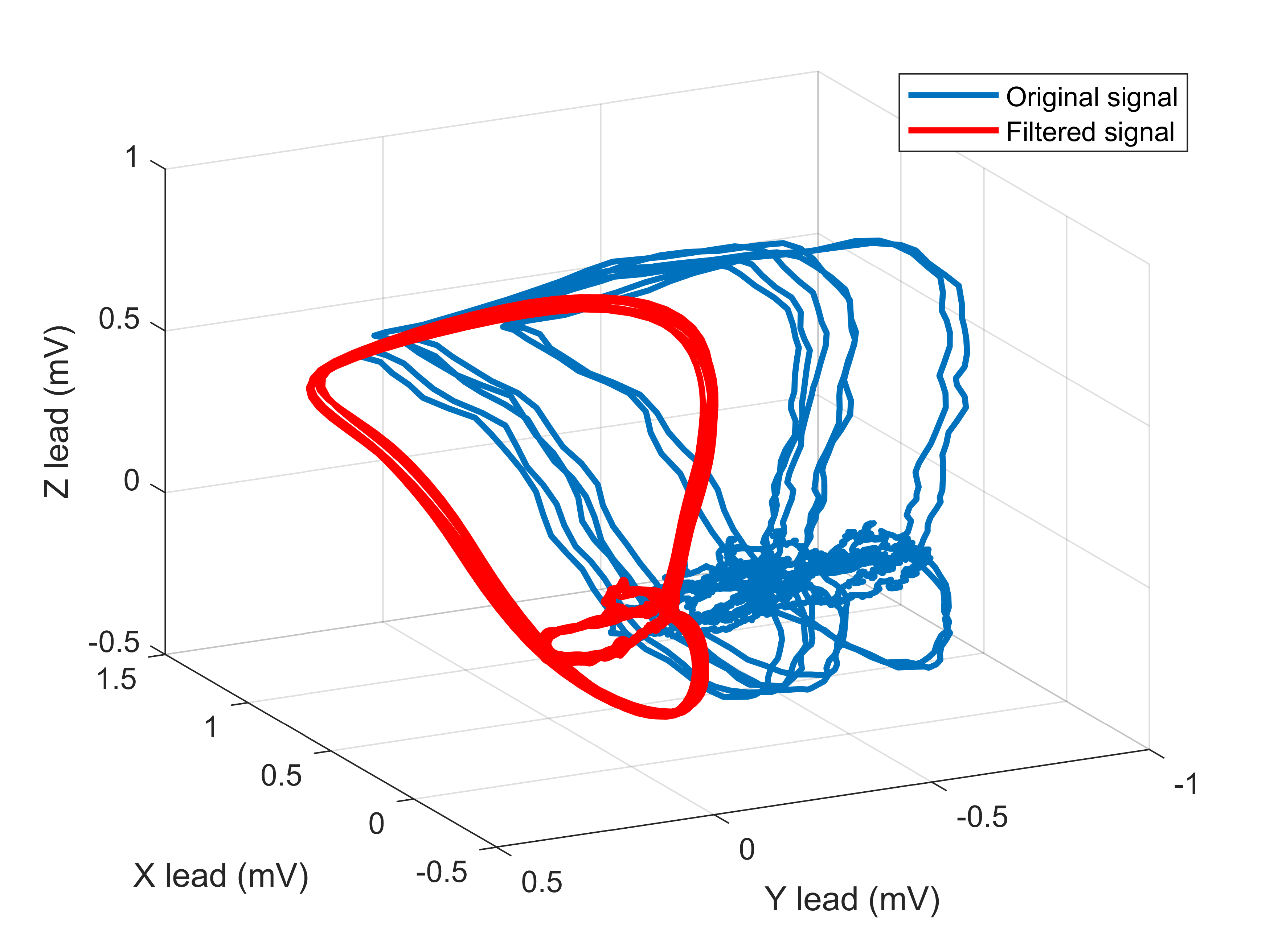

Supplement: Supplementary file 9 [file Image8.PNG]

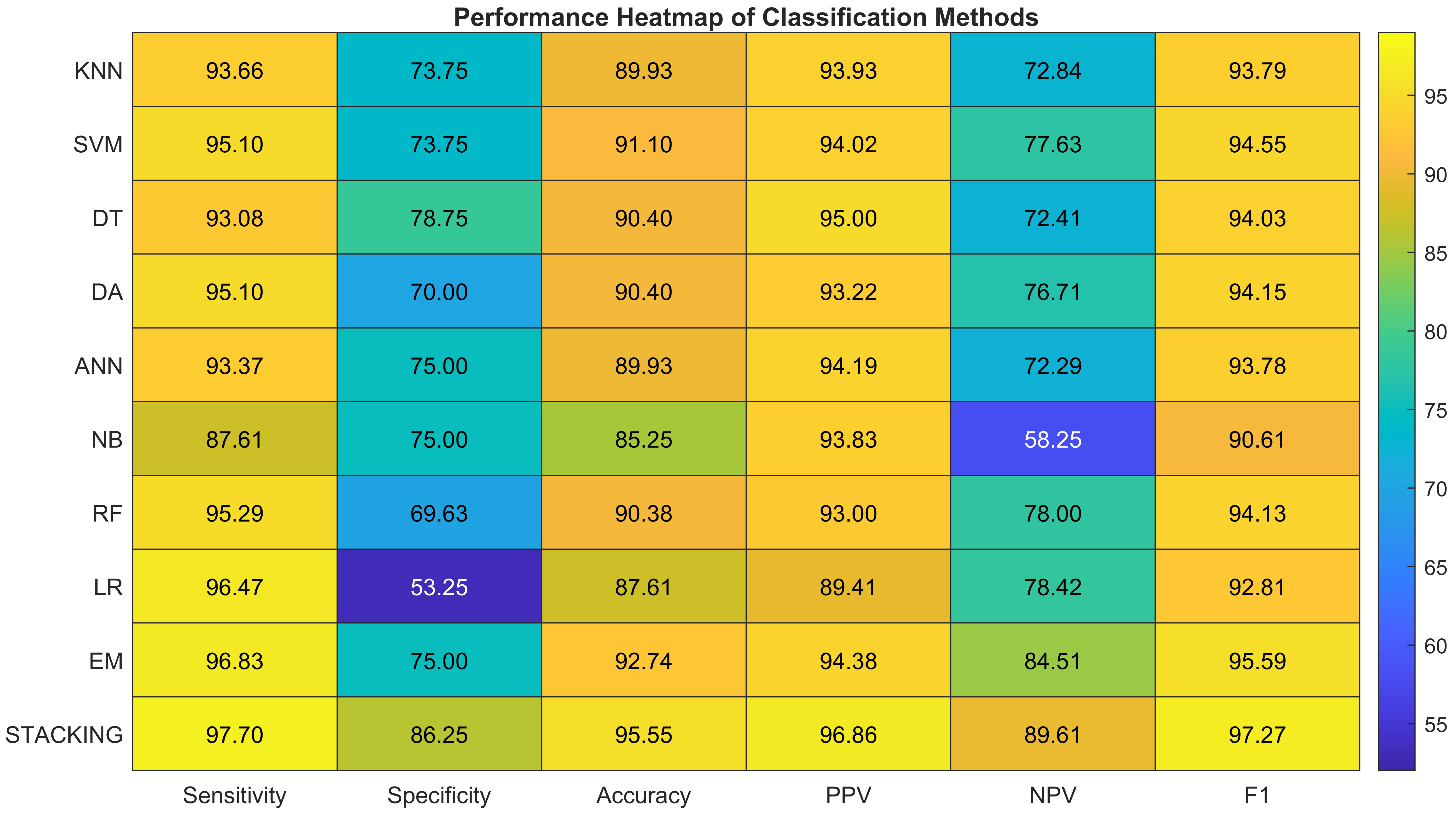

Supplement: Supplementary file 10 [file Image9.PNG]

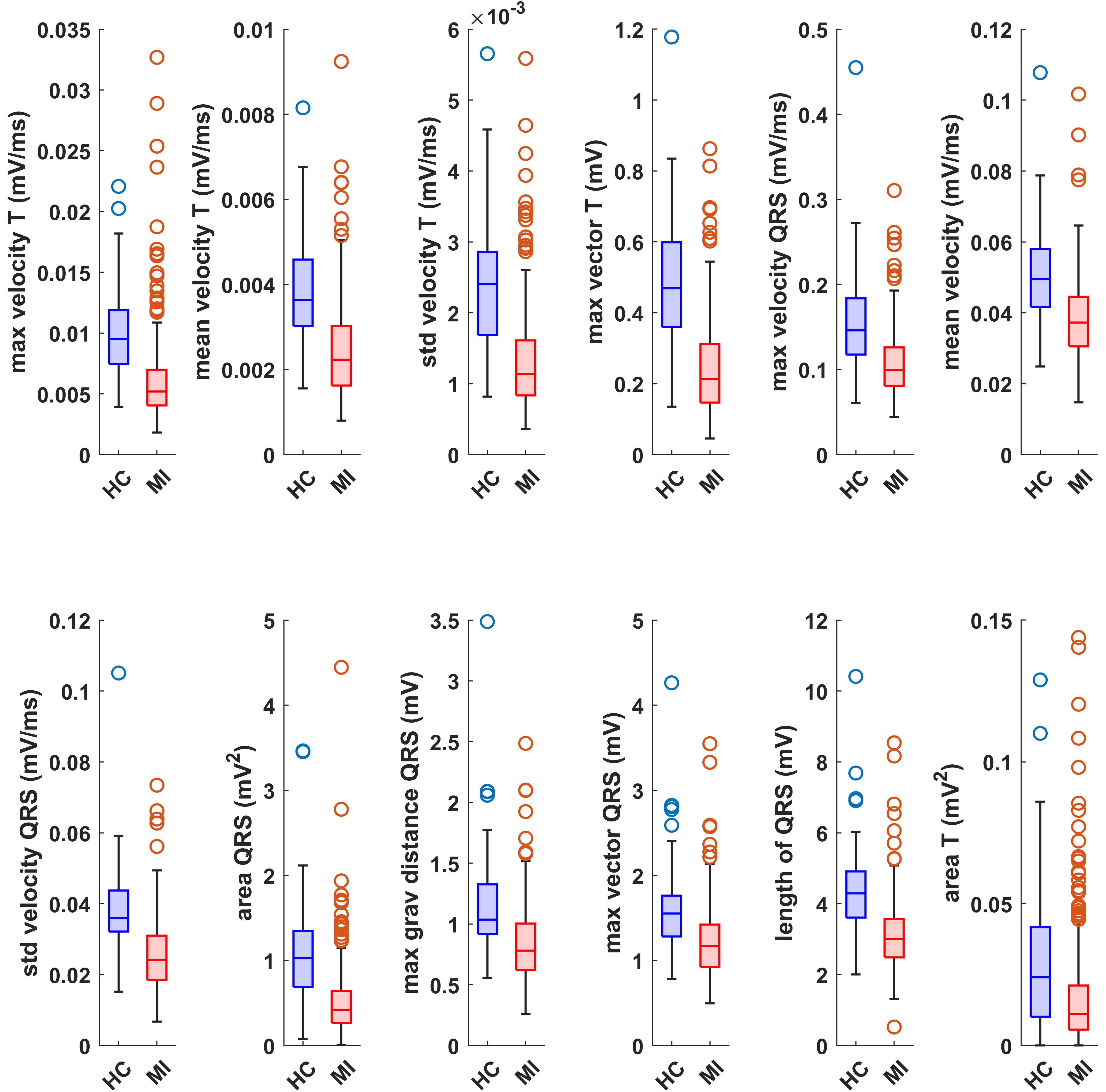

Supplement: Supplementary file 11 [file Image6.PNG]

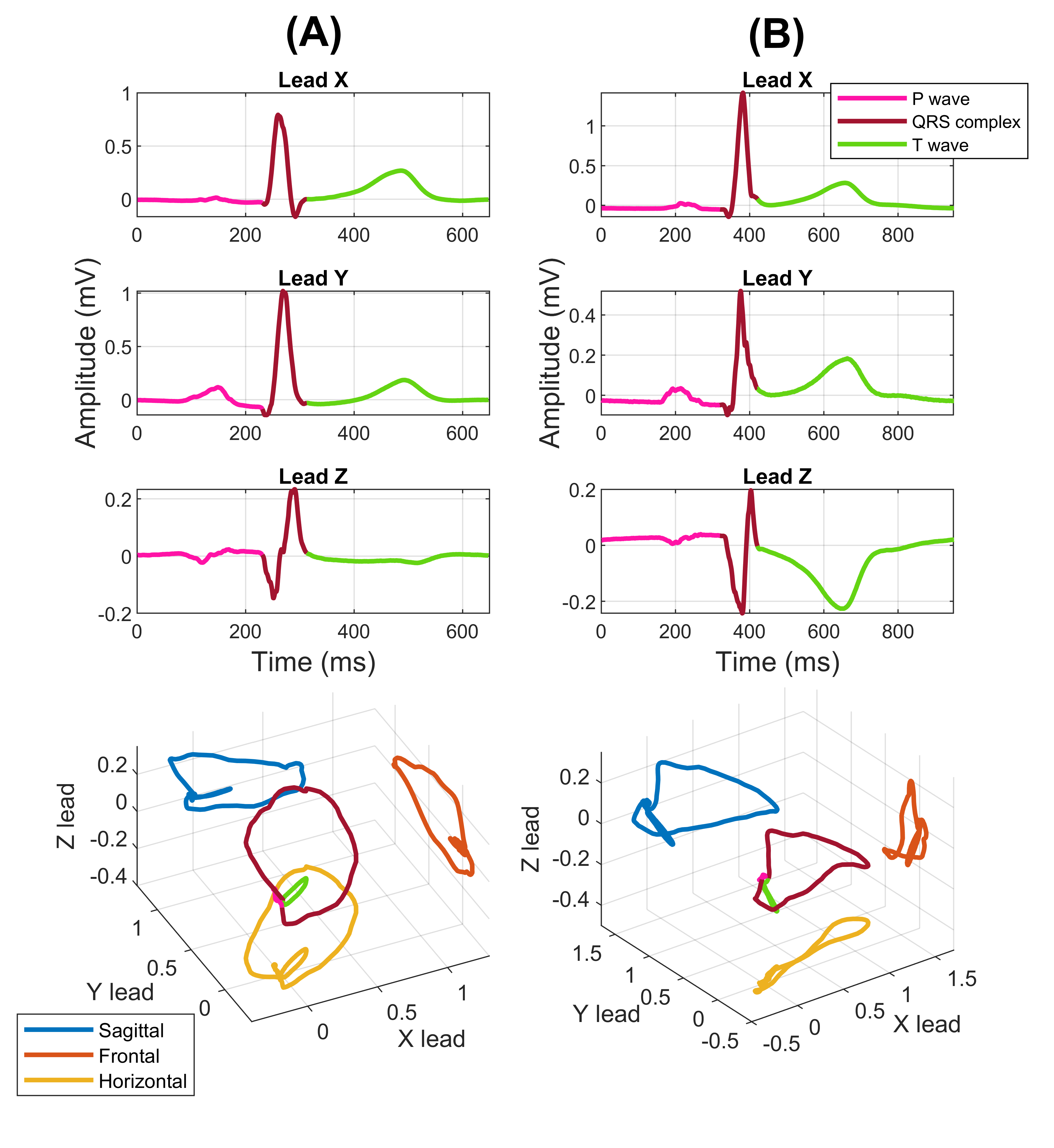

Supplement: Supplementary file 13 [file Image10.PNG]
