# Supplementary material for: Enhancing myocardial infarction detection with vectorcardiography: fusion-based comparative analysis of machine learning methods
Source: Front Physiol. 2026 Jan 5;16:1683956. doi: 10.3389/fphys.2025.1683956 (PMC12813126; doi:10.3389/fphys.2025.1683956)
Supplement: Supplementary file 5 [file DataSheet1.pdf]

1 APPENDIX ALGORITHM

Algorithm 1: VCG preprocessing and feature extraction pipeline.

Input: Raw Frank VCG signals $X(t)$, $Y(t)$, $Z(t)$, sampling rate f_s
Output: Feature matrix F (patients $\times$ 12 features)

1. Baseline removal:
Apply second-order Savitzky–Golay filter ($window = 1201$) to each lead.
Subtract estimated baseline $\Rightarrow$ obtain X_b, Y_b, Z_b.

2. Vector magnitude and R-peak detection:
Compute $VM(t) = \sqrt{X_b^2 + Y_b^2 + Z_b^2}$ and detect R-peaks.

3. Beat segmentation:
For each R-peak, extract QRS window and T window by:

- Generate a high-frequency signal and count zero crossings per segment.
- QRS complexes correspond to segments with a low number of zero crossings.
- Using an adaptive threshold, determine the onset and offset of each QRS complex.

Reject abnormal beats based on length of QRS.
Same principle for T window.

4. Projection to optimal plane (PCA):
Center QRS and T matrices, perform SVD ($X_c = U\Lambda W^T$).
Use first two columns of W to project points $\Rightarrow$ $(PCA1, PCA2)$.

5. Feature extraction (12 total):

- arcQRS – length of QRS in PCA plane.
- maxVecQRS/T – maximum vector magnitude during QRS/T.
- MaxGravQRS – maximum distance from loop centroid.
- maxVel, meanVel, stdVel for QRS and T loops.
- areaQRS/T – area of loop (shoelace formula).

6. Statistical analysis:
Perform Shapiro-Wilk test (MI and HC) for each feature.
Perform Mann–Whitney test (MI vs. HC) for each feature.

7. Cross-validation and stacking:
Use patient-wise 10-fold CV.
Generate out-of-fold (OOF) predictions from base models for meta-classifier training.
Evaluate performance (Accuracy, Sensitivity, Specificity, PPV, NPV, f1).
